# Engineering the oleaginous red yeast *Rhodotorula glutinis* for simultaneous β-carotene and cellulase production

**DOI:** 10.1038/s41598-018-29194-z

**Published:** 2018-07-18

**Authors:** Hong-Wei Pi, Marimuthu Anandharaj, Yi-Ying Kao, Yu-Ju Lin, Jui-Jen Chang, Wen-Hsiung Li

**Affiliations:** 1 Ph.D. Program in Microbial Genomics, National Chung Hsing University and Academia Sinica, Taipei, Taiwan; 2 Biodiversity Research Center, Academia Sinica, Nankang, Taipei, 11529 Taiwan; 3 Molecular and Biological Agricultural Sciences Program, Taiwan International Graduate Program, National Chung Hsing University and Academia Sinica, Taipei, 11529 Taiwan; 4 Graduate Institute of Biotechnology, National Chung Hsing University, Taichung, 40227 Taiwan; 5 Department of Medical Research, China Medical University Hospital, China Medical University, No. 91 Hsueh-Shih Road, Taichung, 402 Taiwan; 6 Biotechnology center, National Chung Hsing University, Taichung, 40227 Taiwan; 7 Department of Ecology and Evolution, University of Chicago, Chicago, 60637 USA

## Abstract

*Rhodotorula glutinis*, an oleaginous red yeast, intrinsically produces several bio-products (i.e., lipids, carotenoids and enzymes) and is regarded as a potential host for biorefinery. In view of the limited available genetic engineering tools for this yeast, we have developed a useful genetic transformation method and transformed the β-carotene biosynthesis genes (*crtI*, *crtE*, *crtYB* and *tHMG1*) and cellulase genes (*CBHI*, *CBHII*, *EgI*, *EgIII*, *EglA* and *BGS*) into *R*. *glutinis* genome. The transformant P4-10-9-63Y-14B produced significantly higher β-carotene (27.13 ± 0.66 mg/g) than the wild type and also exhibited cellulase activity. Furthermore, the lipid production and salt tolerance ability of the transformants were unaffected. This is the first study to engineer the *R*. *glutinis* for simultaneous β-carotene and cellulase production. As *R*. *glutinis* can grow in sea water and can be engineered to utilize the cheaper substrates (i.e. biomass) for the production of biofuels or valuable compounds, it is a promising host for biorefinery.

## Introduction

*Rhodotorula glutinis* is an oleaginous red yeast capable of producing several valuable compounds including microbial lipids, pigments and enzymes. *R*. *glutinis* contains up to 70% lipids in their dry weight biomass, and is non-toxic and relatively easy to grow and harvest^1^. It has been demonstrated that *R*. *glutinis* is a potential host for biodiesel industry due to the accumulation of polyunsaturated fatty acid triacylglycerol inside the cells, which is similar to vegetable oils^2,3^. Besides the high lipid production, *R*. *glutinis* can produce β-carotene, which is regarded as a valuable compound in healthcare industry and has anti-carcinogenic and antioxidant properties^4,5^. The Business Communications Company Research reported that β-carotene has the largest share in global carotenoids market (more than $300 million by 2018)^6^. So the demand of β-carotene is increasing. However, concentration of β-carotene in vegetable (e.g. carrot, 0.02 mg/g) is low and is decreased during transfer and storage^7^. Therefore, synthesizing β-carotene by microbes is an ideal approach. *R*. *glutinis* is a well-known β-carotene producing yeast in the industry^5^. Although there were many studies focused on different hosts, *R*. *glutinis* can utilize various low-cost carbon sources, making it an attractive candidate for producing lipids and β-carotene in industries^5,8^. Several researchers have used crude glycerol as a substrate for *R*. *glutinis* to produce microbial lipids^9^. Moreover, *R*. *glutinis* is successfully cultivated in brewery effluents^10^. Therefore, *R*. *glutinis* is a promising biorefinery host for the production of microbial lipids and β-carotene using cheap substrates as a carbon source^11^.

Previously, several studies have demonstrated microbial lipid and β-carotene production using wild type *R*. *glutinis*^3^. In order to reduce the high fermentation cost of *R*. *glutinis*, one may increase the productivity of valuable products and/or use cheaper feedstock for fermentation (i.e. biomass, waste water, effluents, etc.). Previous researchers focused on improving the carotenoid production ability of *R*. *glutinis* by optimizing the fermentation conditions (i.e. temperature, pH and dissolved oxygen) or optimizing the ratios of carbon or nitrogen sources^12,13^. Furthermore, *R*. *glutinis* was subjected to mutagenesis using UV irradiation or chemical mutagens to improve its carotenoid production and the mutant strain could produce higher amounts of β-carotene using sea water as a substrate^14–17^. Lignocellulosic biomass, which is the most available, cheap and renewable source in nature, makes it becoming an idea carbon sources for clean energy or bio-products. Lignocellulosic biomass is abundant and renewable in nature and regarded as a potential feedstock for fermentation. However, conversion of biomass into fermentable sugars requires at least three types of cellulases, including endoglucanase (EG), exoglucanase (cellobiohydrolase, CBH), and β-D-glucosidase (BGS)^18,19^. More recent studies revealed that some *R*. *glutinis* strains contain endoglucanases and possess wheat or rice straw degradation ability^5,20,21^.

So far, Agrobacterium-mediated transformation (ATMT) has been used to engineer *Rhodotorula* (teleomorph is *Rhodosporidium*) *toruloides*. Lin *et al*. conducted the genomic insertion of multiple antibiotic resistance genes by ATMT^22^. Johns *et al*. investigated four different promoters that could be induced or repressed by different carbon sources^23^. Moreover, Zhang *et al*. transformed three lipid biosynthesis genes into *R*. *toruloides* and improved the lipid production by fourfold^24^. A review article has pointed out the potential engineering processes in *R*. *toruloides*^25^.

Recently, a few studies have engineered *Yarrowia lipolytica*, which is an oleaginous yeast with cellulolytic ability to convert lignocellulosic substrates to lipid^19,26^. To produce β-carotene at a large scale, previous studies engineered *Saccharomyces cerevisiae* and *Y*. *lipolytica* using a synthetic biology approach^27–30^. However, none of the studies focused on developing a bio-refinery host to convert the lignocellulosic substrates to β-carotene and lipid. This is a first study, which focus on engineering the carotenoid pathway along with cellulolytic enzymes in *R*. *glutinis*. In this study, we used synthetic biology tools to improve the β-carotene production and install cellulolytic ability in *R*. *glutinis* by transforming β-carotene biosynthesis pathway genes (*tHMG1*, *crtI*, *crtE* and *crtYB*) and cellulase genes (*CBHI*, *CBHII*, *EgIII*, *EgI*, *EglA* and *BGS*) into its genome. Moreover, we demonstrated the unexpected link between carotenoids and cellulases that suggests the possibility to develop a cell factory to turn biomass wastes into valuable products or/and renewable energy.

## Results

### Establishing a heterologous gene expression platform in *R*. *glutinis*

The result of minimal inhibitory concentration (MIC) (Supplementary Table S1) showed that the *R*. *glutinis* wild type cannot grow in neither 100 nor 200 μg/ml concentration of any of the three antibiotics tested (Zeocin, G418 and Hygromycin), so either concentration can be used to select the transformants. For initial transformation, three gene expression cassettes (Table 1) including phytoene desaturase gene (*crtI*), cellobiohydrolase (*cbhI*) and G418 selection marker gene (*KanMx*) were integrated into the *R*. *glutinis* genome, using frozen protoplast and lithium acetate competent cells^18,31^. The transformants were screened using YP2D supplemented with G418 (200 μg/ml) and the wild type without expression cassette was used as a control. A total of 200 transformants were randomly selected and subcultured for 3 generations to select stable transformants (Supplementary Fig. S1). Then transformants containing both *crtI* and *cbhI* were validated by PCR using the gene specific primer pairs (Supplementary Table S2). Due to the high GC content of *R*. *glutinis*, the PCR amplification of specific genes from their genomic DNA was found to be a difficult task^22,32^. Hence, the genes were amplified using long-range primer pairs and modified touchdown PCR conditions and the wild type was used as a negative control. The results showed that P4-10-9 was successfully transformed and contained the *crtI*, *cbhI* and *KanMx* genes (Supplementary Fig. S2). This is the first study to integrate both β-carotene biosynthesis genes and cellulase genes into the *R*. *glutinis* genome by electroporation. Our gene cassettes were flanked with the specific homologous recombination (HR) region at both ends but we could not confirm the inserts in those regions (data not shown). Previous studies suggested that *Rhodotorula* had a low HR efficiency^33,34^. This suggests that the genes were integrated into the *R*. *glutinis* genome by non-homologous end joining (NHEJ). In future, we may improve the targeting frequency or HR by inhibiting or deleting the NHEJ pathway. Furthermore, *R*. *glutinis* was successfully transformed by the ATMT method to integrate the hygromycin resistance gene (Supplementary Fig. S3). Previous studies applied ATMT in some recalcitrant transformation hosts including *R*. *glutinis*^22,35^. However, ATMT is not suitable for the integration of multiple genes at the same time.

**Table 1:** The gene expressing cassettes used in this study.

Gene	Species	Function	Promoter	terminator	Cassette size (bp)
*crtE*	*Xanthophyllomyces dendrorhous*	Geranylgeranyl pyrophosphate synthase	ScGapDH	ScGapDH	2,083
*crtI*	*Xanthophyllomyces dendrorhous*	Phytoene desaturase	ICL	35 S	3,935
*crtYB*	*Xanthophyllomyces dendrorhous*	Phytoene synthase/lycopene cyclase	ScPGK	ScPGK	3,186
*tHMG1*	*Kluyveromyces marxianus*	Truncated 3-hydroxy-3-methylglutaryl-coenzyme A reductase	ScADHI	ScADHI	2,911
*CBHI*	*Trichoderma reesei*	Cellobiohydrolases	KlADHI	ScGapDH	2,680
*CBHII*	*Trichoderma reesei*	Cellobiohydrolases	KlPGK	ScPGK	2,502
*EgIII*	*Trichoderma reesei*	Endo-β-1,4-glucanases	ScGapDH	ScGapDH	2,161
*EgI*	*Aspergillus niger*	Endo-β-1,4-glucanases	ScPGK	ScPGK	2,731
*EglA*	*Aspergillus niger*	Endo-β-1,4-glucanases	Lac4	Lac4	2,925
*BGS*	*Neocallimastix patriciarum*	β-glucosidases	ScADHI	ScADHI	3,691
*KanMx*	—	Geneticin (G418) resistance	KlGapDH	ScGapDH	1,855
*Sh ble*	—	Zeocin resistance	ScADHI	ScADHI	1,786
*hph*	—	Hygromycin B resistance	ScADHI	ScADHI	2,437
				Total size:	34,890

### Improvement of β-carotene production by installing genes into *R*. *glutinis* genome

**Figure 1:**
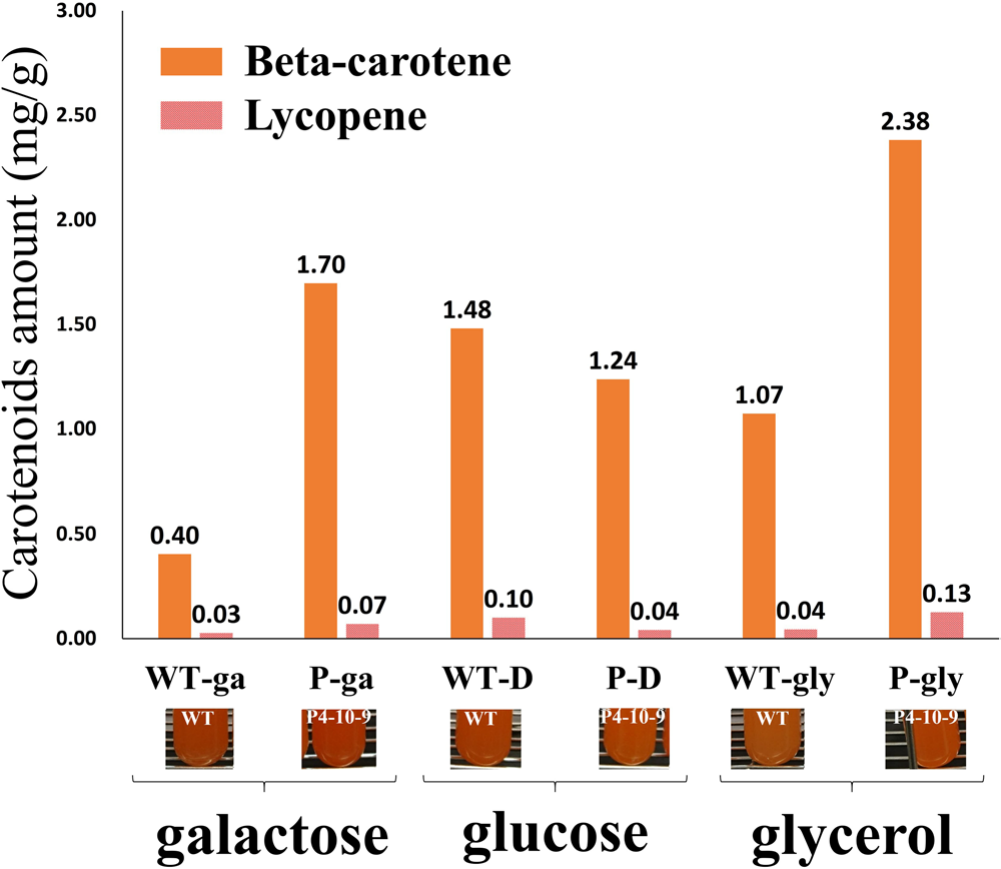
Carotenoid amounts of the P4-10-9 transformant in different carbon sources. The number at the top of a vertical bar shows the β-carotene or lycopene amounts compared to the amount in the wild type.

Based on a previous study^9^, we tested different carbon sources to compare the β-carotene production between wild type and P4-10-9. Three different carbon sources were tested: 2% glucose, 2% galactose and 2% glycerol. The HPLC data showed that P4-10-9 produced more carotenoids than the wild type using galactose or glycerol as a carbon source, compared to glucose (Fig. 1). Similar results were obtained by Johns, Love and Aves^23^, who found that the ICL promoter was inhibited by glucose. It might be because the *crtI* gene was driven by the ICL promoter, so P4-10-9 could produce more carotenoids.

**Figure 2:**
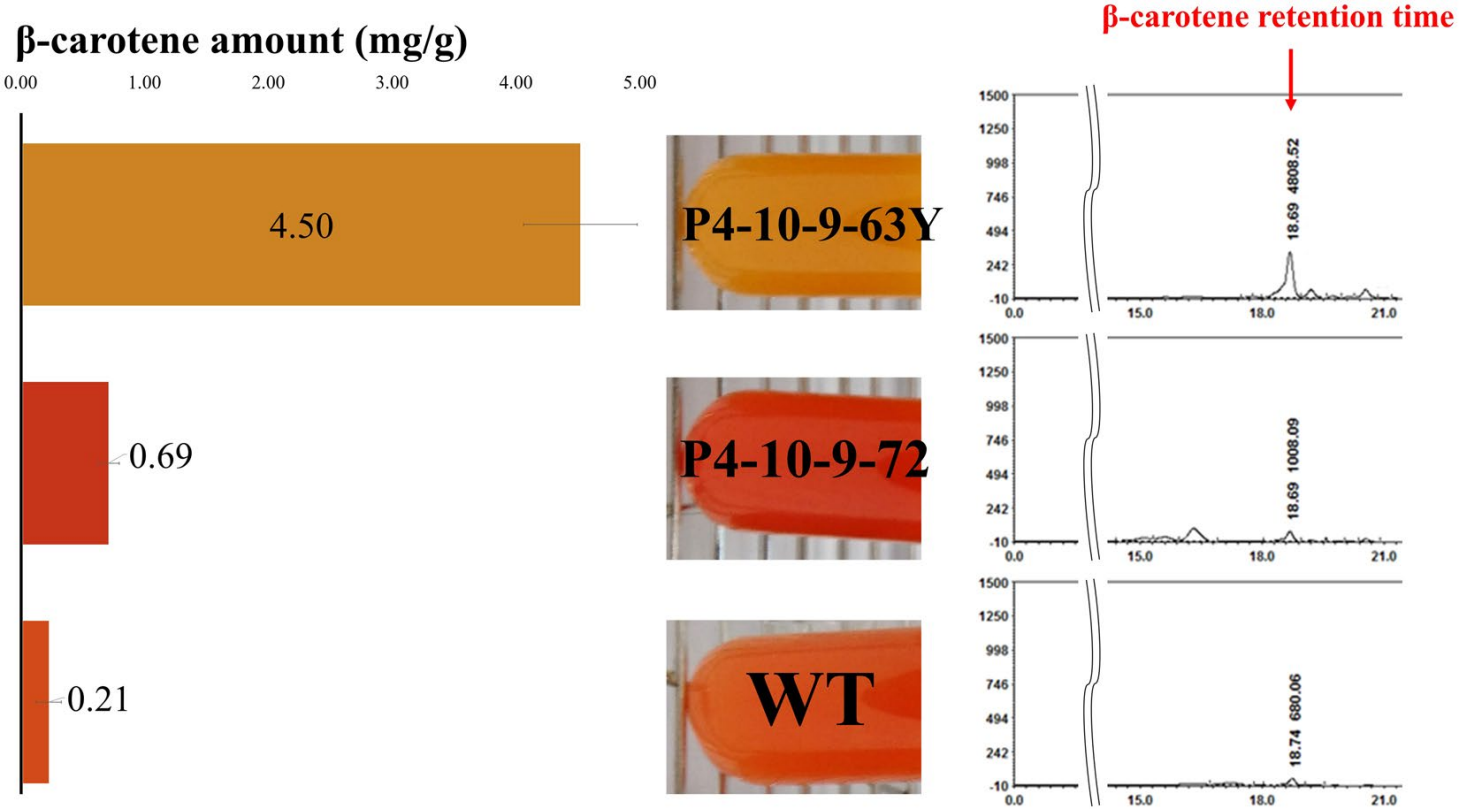
The amounts of β-carotene (mg/g) in P4-10-9-63Y, P4-10-9-72 and the wild type. In the HPLC data, the numbers of sample peaks represent retention times of β-carotene and peak area.

To further improve the β-carotene production, additional functional genes including *crtYB* and *crtE* were integrated into the P4-10-9 genome using electroporation, and transformants were screened using zeocin. 72 transformants were selected and subcultured for 3 generations to obtain stable transformants. The genomic integration of *crtYB* and *crtE* was confirmed by PCR using long-range gene-specific primer pairs (Supplementary Table S2). The PCR amplification (Supplementary Fig. S4) confirmed the integration of *crtE* in transformant 72 and it was named as P4-10-9-72. Similarly, transformant 63 was integrated only with *crtYB* and named as P4-10-9-63Y. To confirm the β-carotene production improvement, transformants P4-10-9-63Y and P4-10-9-72 and wild type *R*. *glutinis* were cultured in 10 ml YP2Gly for 2 weeks and total carotenoids were extracted. The HPLC data showed that both transformants produced higher β-carotene than the wild type (Fig. 2). Especially, β-carotene production of P4-10-9-63Y was improved by 20 folds (4.50 ± 0.46 mg/g), thus confirming the importance of *crtYB* in β-carotene biosynthesis. However, the HPLC data of P4-10-9-72 showed a small β-carotene peak at around the 18 min retention time and several new peaks were observed between 15-17 min (Fig. 2), which might be due to the production of torulene and torularhodin. Indeed, Frengova and Beshkova^36^ demonstrated the production of torulene and torularhodin by *R*. *glutinis*. Furthermore, transformant P4-10-9-72 showed red color and previous studies found that torulene and torularhodin showed characteristic red color^37,38^. Transformant P4-10-9-72 contains both *crtI* and *crtE*, which might improve the precursor availability for the torulene and torularhodin pathways^39,40^. The higher β-carotene producing transformant P4-10-9-63Y showed yellow color. Similarly, Bhosale and Gadre observed yellow color in *R*. *glutinis* after UV treatment, which produced increased β-carotene compared to the parent strain^41^. Hence, the higher β-carotene producing P4-10-9-63Y was selected as a host for further study.

**Figure 3:**
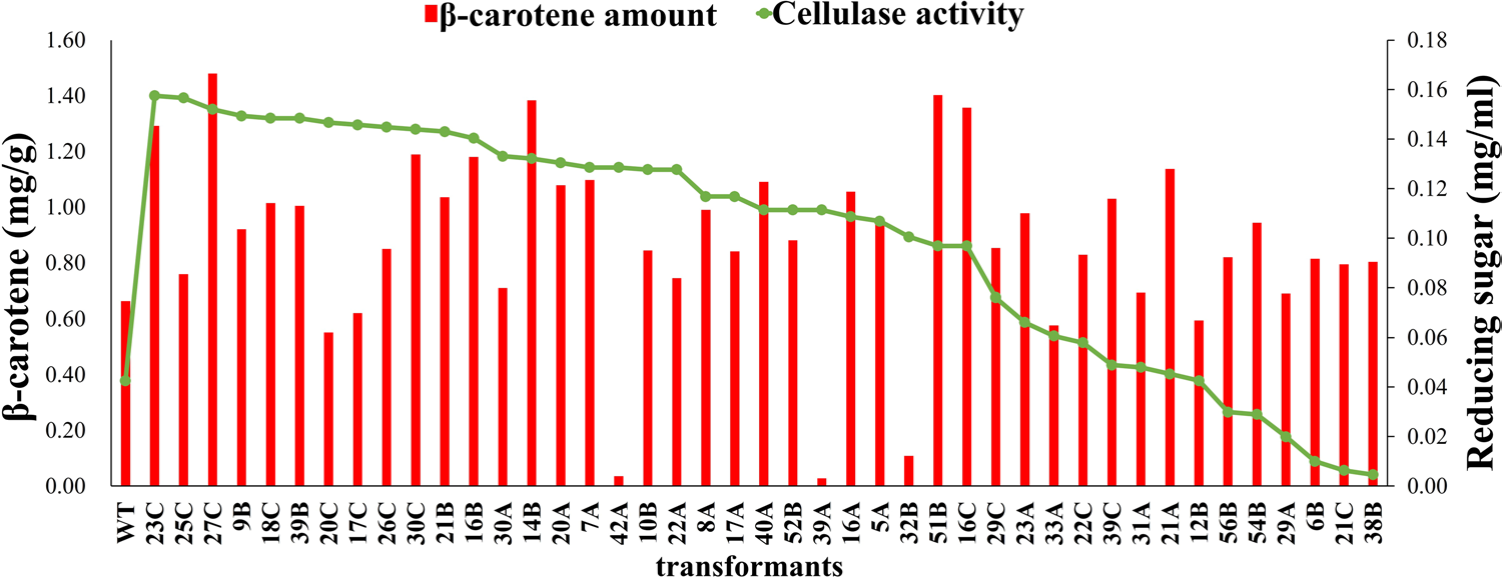
Functional screening of *R*. *glutinis* 43 transformants that contained all the 10 genes.

**Figure 4:**
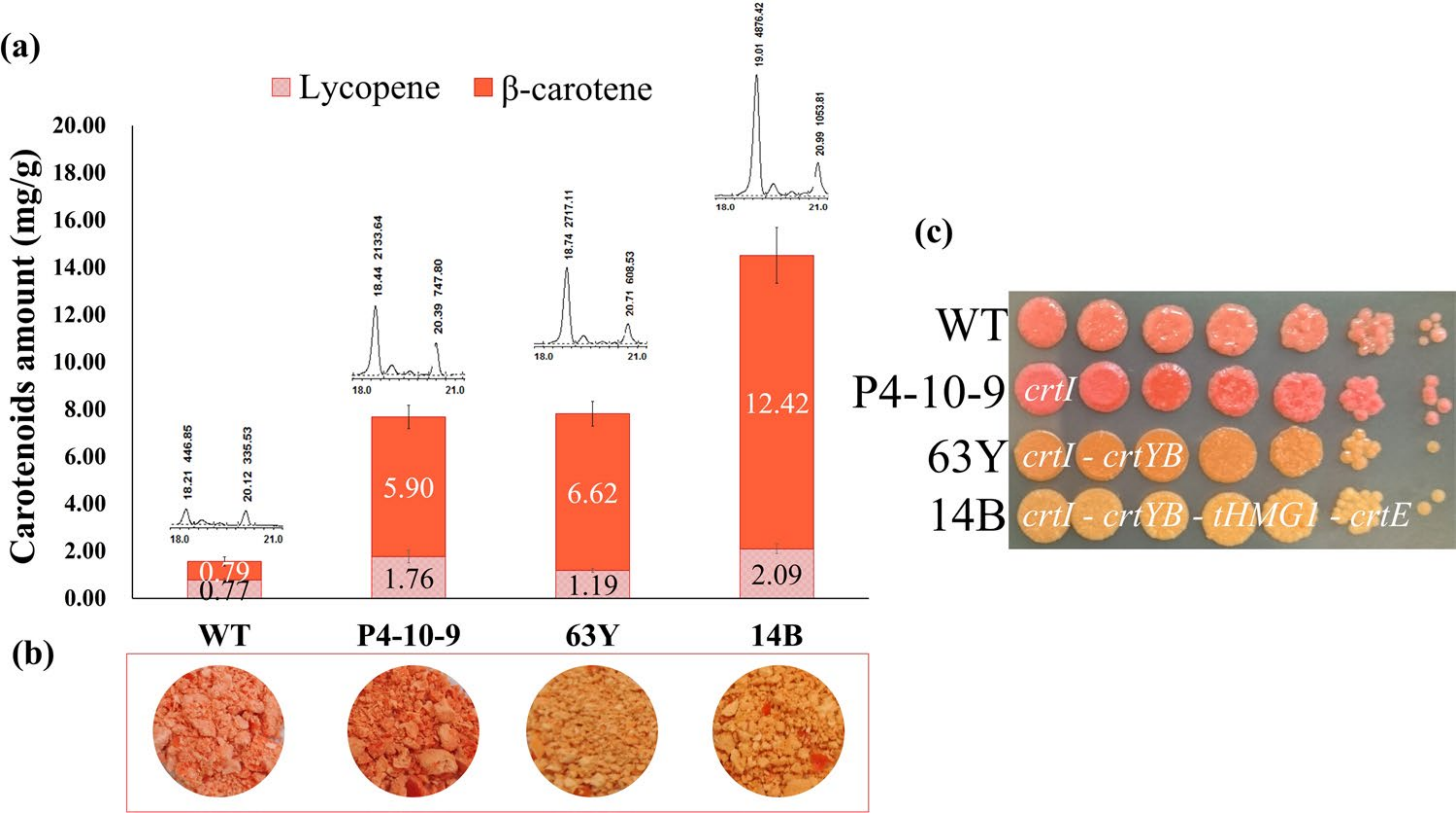
The amounts of carotenoids (mg/g) in different *R*. *glutinis* transformants. (**a**) HPLC analysis. (**b**) The dry biomass before extraction. (**c**) Colony colors on YP2G2Gly agar plates.

The β-carotene production of transformant P4-10-9-63Y was further improved by transforming the upstream genes (*tHMG1* and *crtE*) of the β-carotene biosynthetic pathway. A total of 146 transformants were selected on YP2D plates with hygromycin as a selective marker and subcultured for 3 generations. The PCR results (Supplementary Fig. S5) showed that 43 transformants containing all the four β-carotene biosynthesis pathway genes and selected for functional assays. These 43 transformants could be stably subcultured on selective plates supplemented with G418, zeocin and hygromycin. The β-carotene production of 43 transformants were analyzed and almost every transformant showed improved β-carotene production (Fig. 3). We obtained transformants containing different gene arrangements (Table 2). P4-10-9, P4-10-9-63Y and P4-10-9-63Y-14B were selected for further analysis because they contained different arrangements of *tHMG1*, *crtI*, *crtE* and *crtYB*. We measured the lycopene and β-carotene amounts and observed the color of colonies of transformants compared to the wild type (Fig. 4). We reduced the growing time to avoid the decrease of β-carotene amount and used two carbon source according to Fig. 1. Therefore, we used 10 ml YP2G2Gly and cultured for 7 days. The transformant P4-10-9-63Y produced 8.4 fold higher β-carotene with a significant color change than the wild type (Table 2, Fig. 4b,c). Therefore, the bi-functional enzyme *crtYB* (phytoene synthase and lycopene cyclase) appears to play an important role in the carbon flux from lycopene to β-carotene. In contrast, P4-10-9-63Y-14B contains *tHMG1*, *crtI*, *crtE* and *crtYB*, which improved the conversion of HMG-CoA to β-carotene. The engineered *R*. *glutinis* strain P4-10-9-63Y-14B produced 15.7 fold β-carotene and 2.7 fold higher lycopene compared to the wild type. This is the first study that showed a significant improvement of carotenoid production in the oleaginous red yeast.

**Table 2:** *R*. *glutinis* transformants containing different β-carotene biosynthesis genes.

*R. glutinis* strains	transformed carotenoid biosynthesis genes	Fold of carotenoid increase	
		Lycopene	β-carotene
Wild type	—	—	—
P4-10-9	*crtI*	2.3	7.4
P4-10-9-63Y	*crtI - crtYB*	1.5	8.4
P4-10-9-63Y-14B	*crtI - crtYB - tHMG1 - crtE*	2.7	15.7

### Installation of cellulase ability in *R*. *glutinis*

To increase the value of *R*. *glutinis* as a host for biorefinery applications, the strain P4-10-9-63Y was selected as a host to be transformed with the cellulase genes to utilize the cellulosic biomass. Three types of cellulases (*CBHI*, *CBHII*, *EgIII*, *EgI*, *EglA* and *BGS*) were transformed into the *R*. *glutinis* genome according to our previous studies with other hosts^18,42,43^. The genomic integration of each gene was confirmed by PCR using gene specific primer pairs (Supplementary Table S2, 9–20). The PCR data (Supplementary Fig. S5) showed that several transformants contained all the cellulase genes. We first utilized mix colonies PCR to validate *CBHII*, *EgIII*, *EglA* and *BGS* genes and the results showed correct gene sizes as the positive control. Second, we tested *CBHI* gene by the single colony PCR and selected transformants containing the correct gene size. Then we further confirmed *EgI* in 43 transformants. The cellulase activity was screened using fast-cellulase screening (4% Sigmacell cellulose type 20 as the carbon source) and the data showed that almost all transformants had higher cellulase activity compared to the wild type strain (Fig. 3).

### Demonstrating the biorefinery potential of the engineered *R*. *glutinis*

**Figure 5:**
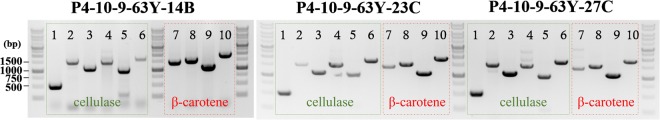
Validation of the 10 genes in *R*. *glutinis* candidate transformants. *The numbers in the figure represent: 1: *eglA* (503 bp), 2: *egI* (1478 bp), 3: *egIII* (1026 bp), 4: *cbhI* (1441 bp), 5: *cbhII* (1002 bp), 6: *BGS* (1690 bp), 7: *tHMG1* (1376 bp), 8: *crtI* (1489 bp), 9: *crtE* (1082 bp) and 10: *crtYB* (1821 bp). The size of amplicons were according to the designing specific primer pairs in Supplementary Table S2.

**Figure 6:**
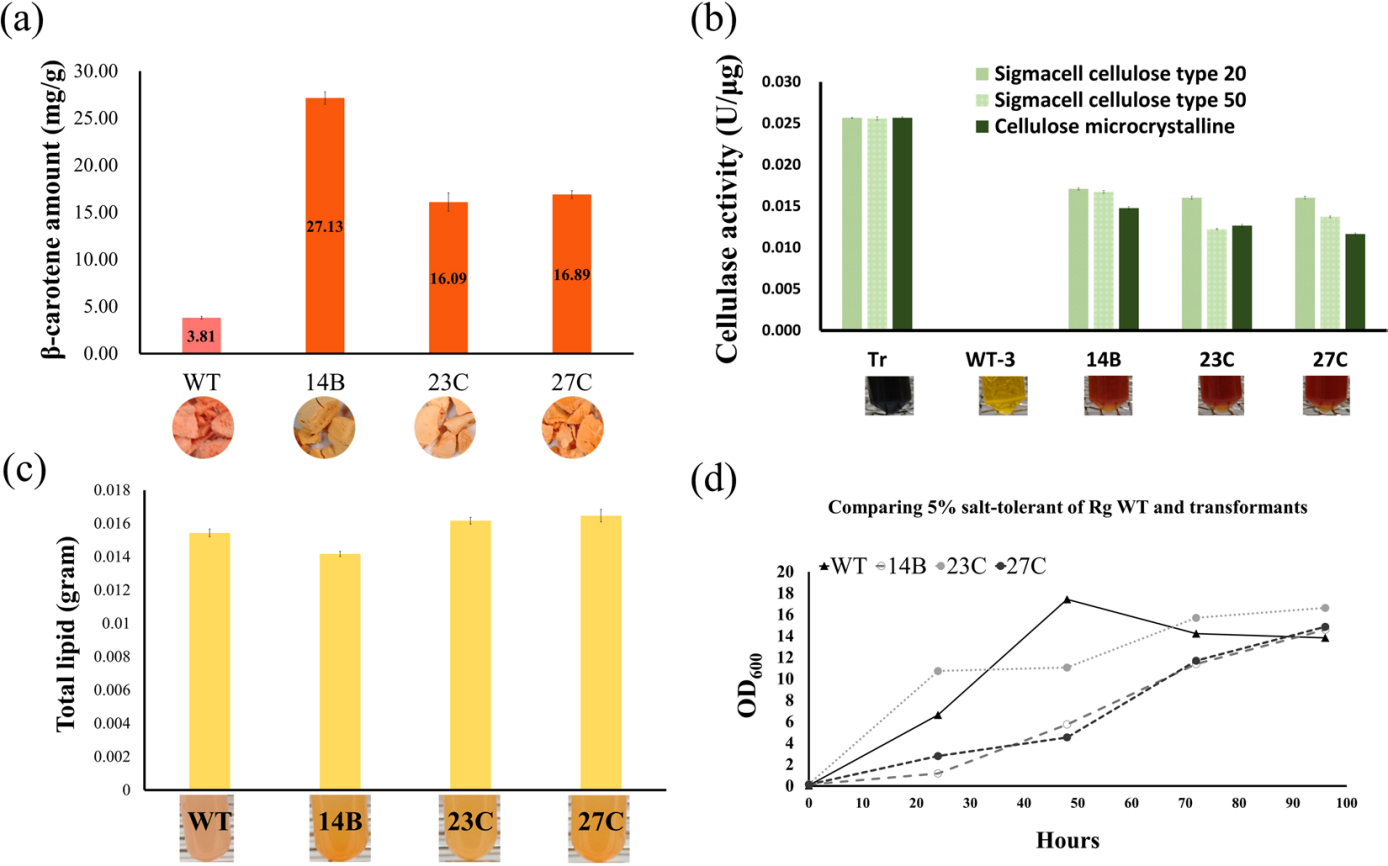
Performances of *R*. *glutinis* candidate transformants. (**a**) Comparison of maximum β-carotene amounts in different transformants. (**b**) Total cellulase activity assay. (**c**) Total lipid weight. (**d**) Salt-tolerance.

The engineered *R*. *glutinis*, P4-10-9-63Y-14B, −23C and −27C were selected based on their genotype (Fig. 5) and phenotype (Fig. 3). The four major properties, i.e., maximum β-carotene amount, total cellulase activity, total lipid and salt-tolerance of transformants, were analyzed. To analyze the maximum β-carotene amounts in selective transformants, we reduced medium from 10 ml to 7 ml because we speculated that the dissolved oxygen might improve the β-carotene amount. Therefore, the selective transformants were cultured in 7 ml YP2G2Gly for 1 week to analyze the maximum β-carotene amounts. Figure 6a showed the β-carotene amounts of selective transformants were increased compared to the wild type. The β-carotene amount of P4-10-9-63Y-14B was improved up to 27.13 ± 0.66 mg/g. To the best of our knowledge, this is the highest β-carotene amount ever produced by *R*. *glutinis*.

To investigate the total cellulase activities of P4-10-9-63Y-14B, −23C and −27C, we tested different cellulosic substrates including Sigmacell cellulose type 20, Sigmacell cellulose type 50 and cellulose microcrystalline. The 50-fold condensed supernatants of *R*. *glutinis* wild type, 14B, 23C and 27C were harvested and the same total protein concentration was used for the total cellulase activity assay^18^. Figure 6b and Supplementary Fig. S6 shows that we have successfully improved the total cellulase activity of *R*. *glutinis* by transforming these cellulase genes. The results showed that the total cellulase activities of 14B (0.017 U/μg), 23 C (0.016 U/μg) and 27 C (0.016 U/μg) were only around 1.53- to 1.63-fold lower than the commercial enzyme Celluclast 1.5 L (0.026 U/μg) in Sigmacell cellulose type 20 substrate. This is the first study to engineer the cellulase genes into *R*. *glutinis* and significantly improved its total cellulase activities. Thus, the engineered *R*. *glutinis* strain might have potential to use the cellulosic substrate as a carbon source to produce a higher amount of β-carotene compared to the wild type (Supplementary Fig. S7).

In order to make sure that the insertion of additional genes did not affect the intrinsic characteristics of *R*. *glutinis*, the total lipid content and salt-tolerance of 14B, 23C and 27C was analyzed. The total lipid weight indeed showed no significant difference between the wild type and transformants (Fig. 6c); the total lipid content was around 70% lipid yield of dry weight biomass, the same as described in a previous study^44^. We also tested the growth of the wild type and transformants in YP2D with 5% NaCl to study their salt tolerance. The results showed that all of the strains were able to grow well in YP2D with 5% NaCl (Fig. 6d). Although the initial growth of the wild type was faster than the transformants, all the strains reached similar OD_600_ after 3 days culturing. We could not observe any significant growth difference between the wild type and transformants in YP2D (data not shown). Moreover, the total lipid content of both the wild type and the transformants were unaffected. So we suggest that the growth rate was not affected significantly. These results demonstrated that engineering the *R*. *glutinis* using our method did not strongly affect the lipid production and salt tolerance of *R*. *glutinis*.

## Discussion

We have successfully integrated multiple gene expression cassettes including 4 β-carotene biosynthesis pathway genes and 6 cellulase genes into the genome of oleaginous red yeast *R*. *glutinis*. The transformant P4-10-9-63Y-14B produced up to 27.13 ± 0.66 mg/g of β-carotene amount. This was achieved by transforming four functional genes (*tHMG1*, *crtI*, *crtE* and *crtYB*) into the *R*. *glutinis* genome. A previous study achieved a similar result in *Saccharomyces cerevisiae*^27^. Although Li *et al*.^30^ discovered several novel β-carotene improving genes, the β-carotene amount (5.9 ± 0.1 mg/g) produced in their engineered *S*. *cerevisiae* was still as low as that in Verwaal *et al*. (2007). Larroude *et al*. applied synthetic biology tools to engineer oleaginous yeast *Yarrowia lipolytica* to obtain the highest β-carotene producing yeast (89.6 mg/g)^29^. These researchers pointed out that β-carotene may be stored and protected in lipid droplets in oleaginous yeast^28^. Gao *et al*. and Larroude *et al*. utilized multiple-copy insertions to improve the β-carotene production. Although there is some difficulty in analyzing the copy number in our *R*. *glutinis* transformants (i.e. RNA extraction), the strategy in this study can improve β-carotene production by 7 to 15 folds. The highest β-carotene amount (DCW) in previous studies were found in *Saccharomyces cerevisiae* (18 mg/g)^45^, *Escherichia coli* (72 mg/g)^46^ and *Yarrowia lipolytica* (89.6 mg/g)^29^. The β-carotene production of engineering strain might further improve by fed-batch cultivations in a bioreactor^28,29^. Our best producer transformant 14B is inferior only to *E*. *coli* and *Y*. *lipolytica*. Moreover, we might improve its β-carotene amount by a bioreactor in the future.

We improved the β-carotene production and installed cellulase ability (*CBHI*, *CBHII*, *EgIII*, *EgI*, *EglA* and *BGS*) at the same time. The cellulase from concentrated supernatants of P4-10-9-63Y-14B were only 1.53-fold lower than the commercial enzyme (Celluclast 1.5 L) purified from *Trichoderma reesei*. The commercial cellulases are produced by cellulolytic fungi, such as *Trichoderma reesei* and *Aspergillus niger*. Each host has its own limitation. For example, *Neocallimastix patriciarum* can produce highly efficient β-glucosidase but it is an obligate anaerobic rumen fungus^47^. Therefore, many studies engineered organisms with multiple cellulolytic properties to design an ideal cellulolytic host. Chang *et al*.^43^ engineered a cellulolytic *K*. *marxianus* with the similar cellulase genes as in this study^43^. The total cellulase activity of *R*. *glutinis* can be further improved by synthetic biology strategy. The functional analysis suggested that transformants also maintained its original lipid yield and salt-tolerance ability. To produce β-carotene, we need to culture for 7 days to get the highest β-carotene amounts. Our transformants can reach the same OD600 as wild type after 3 days in YP2D with 5% NaCl. So we suggested that we can produce higher β-carotene amount even in salty condition. We confirmed that the total lipid content of both the wild type and the transformants were unaffected (around 70% lipid yield), thus further supporting our statement. From these experiments, it seems that our engineering strategy is good. Several reports have described the synthesis of carotenoids, cellulases or lipid from fungi. However, in previous studies the hosts were usually engineered to produce a single product. Our goal is to turn cellulolytic wastes into β-carotene and renewable energy by a single host. In conclusion, we have upgraded the potential applications of *R*. *glutinis* for biorefinery using a synthetic biology approach and demonstrated that *R*. *glutinis* is a potential host for biorefinery.

## Materials and Methods

### Strains, media, and growth conditions

The oleaginous yeast *R*. *glutinis* BCRC 22360 (Bioresource Collection and Research Center, Taiwan) was kindly provided by Dr. Hong-Wei Yen (Tunghai University, Taiwan). *R*. *glutinis* was cultured in different growth media at 30 °C with 300 rpm for several days. The basic growth media contained 1% Yeast Extract (BactoDifco) and 1% Peptone (BactoDifco) as a nitrogen source and 2% dextrose (2D), 2% galactose (2 G) or 2% glycerol (2Gly) as a carbon source. For screening the *R*. *glutinis* transformants, a previously described medium was used^48^. The lipid inducing media (70 g/L glucose, 0.75 g/L yeast extract, 1.7 g/L yeast nitrogen base without amino acids and ammonium sulfate, and 0.1 g/L (NH_4_)_2_SO_4_, pH 5.6) were applied for analyzing the total lipid content in *R*. *glutinis*^24^.

### Establishing a heterologous gene expression platform in *R*. *glutinis*

In this study, we intended to develop an efficient transformation tool for *R*. *glutinis*. *R*. *glutinis* electro-competent cells were prepared using two different methods. First, lithium acetate was used to prepare competent cells modified from previous studies for *Kluyveromyces marxianus* and *Rhodotorula gracilis*^18,49^. Briefly, *R*. *glutinis* cells were cultivated in 5 ml YP2D from the single colony at 30 °C with 300 rpm and then 0.2 OD cells were subcultured into 50 ml YP2D until reaching 0.6~1.4 OD. Cells were harvested at 3000 rpm for 3 min (4 °C) and washed with 5 ml ice-cold distilled H_2_O. Then, cells were resuspended in TMLSD buffer (10 mM Tris-HCl buffer (pH 8.0) containing 2 mM MgCl_2_, 100 mM lithium acetate, 270 mM sucrose, 10 mM dithiothreitol) and incubated at room temperature for 1 h. After the incubation, cells were harvested as described above and washed twice with TMS buffer (10 mM Tris-HCl buffer (pH 8.0) containing 2 mM MgCl_2_, 270 mM sucrose). Finally, competent cells were resuspended in TMS buffer and stored in −80 °C.

For the second method, we used the frozen protoplast protocol to engineer *R*. *glutinis*. The *R*. *glutinis* protoplast was prepared according to previous studies^31,50,51^ with some modifications. Briefly, single colonies of *R*. *glutinis* were inoculated in 50 mL of YP2D medium at 30 °C for 15 h. Cells were harvested at 3000 rpm for 10 min and suspended in 20 mL distilled H_2_O. Cells were harvested again as described above and gently resuspended in 10 mL 1 M sorbitol, followed by harvesting and suspending in 10 mL of sorbitol, sodium citrate, EDTA and β-mercaptoethanol (SCEM). Then, cells were mixed with 40 μl of lyticase solution (25,000U/ml) and incubated at 30 °C for 1 h. After the lyticase digestion cells were suspended in SCEM (10^9^ cells/ml), 1 ml cell suspension was added 0.5 ml of lytic enzyme solution (1.5% (w/v) Zymolyase 60,000) for overnight culturing. Cells were centrifuged gently at 300 g for 5 min in round-bottom plastic tubes and suspended in 10 mL 1 M sorbitol by gently tapping the tube. Then cells were centrifuged at 300 g for 5 min, and the supernatant was discarded. This procedure was repeated to remove lyticase thoroughly. Finally, cells were suspended in 2 mL CaST solution (CaCl_2_, sorbitol and Tris-HCl) along with 2 mL cell-storage solution and protoplast cells were stored at −80 °C.

### Electroporation

Electroporation was performed by mixing the 10–15 μl DNA with 50 μl competent cells or protoplasts and kept on ice for 15 min. Then cells were transferred to the ice-cold aluminum cuvette (0.2 cm gap Gene Pulser/MicroPulser Electroporation Cuvettes, Bio-Rad, USA) and electroporation was performed (1.2 kV or 400 V, 400 Ω and 25 μF capacitance), using a MicroPulser (Bio-Rad Laboratories, USA). After electroporation, cells were resuspended in 1 mL ice-cold YP2D and transferred into new tubes on ice for 15 min, and then incubated at 30 °C for 4 h. The cell suspension was spread onto YP2D plates containing selection markers (i.e. Kanamycin (G418), Zeocin or Hygromycin) and incubated at 30 °C for 4–5 days.

### Transforming genes into the *R*. *glutinis* genome

The carotenoid biosynthetic pathway in *Rhodotorula* species has been well studied^3,36^. Generally, *R*. *glutinis* synthesizes β-carotene from the precursor acetyl-CoA (Supplementary Fig. S8). To increase the β-carotene amount in *R*. *glutinis*, the carotenogenic genes including geranylgeranyl pyrophosphate synthase (*crtE*), phytoene desaturase (*crtI*) and phytoene synthase/lycopene cyclase (*crtYB*) from *Xanthophyllomyces dendrorhous* (red yeast) were selected and integrated into the *R*. *glutinis* genome. Similarly, for improving the metabolic flux of carotenoid pathway, a *tHMG1* (a truncated 3-hydroxy-3-methylglutaryl-coenzyme A reductase) gene was selected from *Kluyveromyces marxianus*^27,52^. The carotenogenic genes were in the pUC18 vector and the construction of each gene cassette was as in Chang *et al*.^52^. Briefly, the carotenogenic genes were cloned in between the yeast promoter and terminator using specific restriction enzymes. The gene cassettes were amplified using TransStart FastPfu Fly (Ultra) High-Fidelity DNA Polymerases and the primers pairs were listed in Table S2.

For the simultaneous improvement of β-carotene production and cellulose utilization ability of *R*. *glutinis*, three types of fungal cellulase genes were selected and integrated into the *R*. *glutinis* genome^18,43^. The cellulase genes included two cellobiohydrolases (*CBHI* and *CBHII*, from *Trichoderma reesei*), three endo-β-1,4-glucanases (*EgIII*, from *T*. *reesei*; *EgI* and *EglA*, from *Aspergillus niger*) and β-glucosidases (*BGS*, from *Neocallimastix patriciarum*). Each gene was fused with a secretion signal (α-factor) at the N-terminal for efficient secretion out from the cell. Each gene was flanked with independent inducible promoters and terminators (Table 1). The amplification of six cellulase gene cassettes used the same method as in the carotenogenic gene cassettes. Each cassette was in the pUC18 vector as in previous studies^18,43^. The gene cassettes were integrated using three selection marker genes: G418 resistance gene (neomycin phosphotransferase gene, *KanMx*), Zeocin resistance gene (*Sh ble*) and Hygromycin phosphotransferase gene (*hph*). The MIC of three different selection markers (i.e., Zeocin, G418 and Hygromycin) were tested on *R*. *glutinis* wild type using YP2D plates supplemented with different concentrations of antibiotics.

### Transformant screening

The transformants were sub-cultured for 3 generations to select stable transformants. Then, transformants were mixed with QuickExtract ((QE), DNA Extraction Solution 1.0, Epicentre, USA) for rapid extraction of genomic DNA and used as a template for PCR verification. Each transformed functional genes were confirmed by PCR using the gene specific primer pairs (Supplementary Table S2). Finally, the transformants with integrated gene cassettes were screened for the β-carotene production and cellulase activity.

### β-carotene extraction and Analytical methods

To analyze the β-carotene production in transformants, single colonies were inoculated into 5 ml medium^48^ and incubated at 30 °C, with 300 rpm for 7 days. Cells were harvested by centrifugation (6000 rpm, 10 min), lyophilized and suspended in 1 ml acetone. Cells were then subjected to mechanical disruption using MagNA Lyser (MagNA Lyser Instrument, Roche) at 6,000 rpm, 20 s for 3 times. For screening the transformants, 1 ml acetone was used in crude extraction. To estimate the maximum β-carotene amount of a transformant, the extraction step was repeated until colorless extracts appeared. The carotenoid extracts were analyzed using High-Performance Liquid Chromatography (HPLC) with a Nomura Chemical Develosil C30-UG Column (3 mm, ID 4.6 mm × L 250 mm- UG17346250W, Interlink Scientific Services, UK), using two buffer systems including buffer A: methanol/MTBE/water (81:15:4) and buffer B: methanol/MTBE/water (7:90:3) as mobile phases. The HPLC condition was described in previous studies^52,53^. The commercial free-form β-carotene and lycopene were used as the standards (Sigma–Aldrich Co. LLC, USA).

### Cellulase activity assay

To screen the transformants with higher cellulase activity, a cellulase activity assay was conducted as described in previous studies^18,54^. Single colonies of transformants were incubated in 5 ml YP2D2G for 3 days and cells were harvested and washed twice with PBS. The washed cells were inoculated into 5 ml YP supplemented with 4% Sigmacell cellulose type 20 as the sole carbon source under 30 °C with 300 rpm for 7 days. The initial and final reducing sugar concentrations were analyzed using the Dinitrosalicylic (DNS) colorimetric method^55,56^. Similarly, total cellulase activity was demonstrated using different cellulosic substrates such as Sigmacell cellulose type 20, Sigmacell cellulose type 50 (SigmaAldrich, St.Louis, MO, USA) and cellulose microcrystalline (Merck, Darmstadt, Germany)^54^. For the cellulose degradation assay, the transformants and wild type *R*. *glutinis* were cultured in 50 ml YP2D2G for 3 days and supernatants were concentrated 50-fold using Vivaspin 20 (10,000 molecular weight cut off, PES membrane, GE Healthcare) at 4 °C. The commercial cellulases were used as a positive control, including cellulase powder from *Aspergillus niger* (≥0.3 units/mg solid, Sigma Aldrich C1184, USA) and cellulase liquid from *Trichoderma reesei* (≥700 units/g, Celluclast 1.5 L, Sigma Aldrich C2730, USA). The total protein concentration was determined by Bio-Rad Protein Assay Kit using the bovine serum albumin (BSA) as a standard. The assay reaction contained 32 μg protein, sodium acetate (50 mM, pH = 5) and cellulose substrates (2%, final concentration). The reaction mixture was incubated at 50 °C overnight. The initial and final reducing sugar amounts were analyzed by the DNS method.

### Lipid content and salt tolerance analysis

The total lipid content of *R*. *glutinis* was determined using the method for *Rhodosporidium toruloides* with some modifications^24,57^. Briefly, *R*. *glutinis* wild type and transformants were cultured in 5 ml lipid-inducing medium for 3 days and cells were harvested, frozen and then freeze-dried. Then, the dry-cells were suspended in 1 ml chloroform/methanol (2:1 volumetric) and homogenized using a MagNA Lyser. The homogenized samples were then mixed with 0.2 ml ddH_2_O and vortexed for 15 s. The organic layer was taken using a needle and washed with 0.1 ml NaCl (0.1%, w/v) solution. The extract was repeated until the clear organic layer appeared and was dried in a hood at room temperature overnight followed by 1 h at 80 °C in a pre-weighed tube. The total lipid weight was determined. To determine the salt-tolerance of transformants, 5% NaCl was added to the YP2D media and the growth OD was measured every 24 h in 4 days.

## Electronic supplementary material

Supplemental information

Supplementary raw data

Figure 1 Raw data

Figure 2 raw data

Figure 3 Raw data

Figure 4 Raw data

Fugre 6a Raw data

Figure 6b Raw data

Figure 6c Raw data

Figure 6d Raw data
