# Supplementary material for: Engineering the oleaginous red yeast Rhodotorula glutinis for simultaneous β-carotene and cellulase production
Source: Sci Rep. 2018 Jul 18;8:10850. doi: 10.1038/s41598-018-29194-z (PMC6052021; doi:10.1038/s41598-018-29194-z)
Supplement: Supplementary file 1 — Supplemental information [file 41598_2018_29194_MOESM1_ESM.pdf]

Supplementary Information

Engineering the oleaginous red yeast *Rhodotorula glutinis* for simultaneous β-carotene and cellulase production

Hong-Wei Pi[1, 2], Marimuthu Anandharaj[2, 3, 4], Yi-Ying Kao[2], Yu-Ju Lin[2], Jui-Jen Chang[5, *], Wen-Hsiung Li[2, 6, 7, *]

Affiliations:

[1]Ph.D. Program in Microbial Genomics, National Chung Hsing University and Academia Sinica, Taiwan.

[2]Biodiversity Research Center, Academia Sinica, Nankang, Taipei 11529, Taiwan.

[3]Molecular and Biological Agricultural Sciences Program, Taiwan International Graduate Program, National Chung Hsing University and Academia Sinica, Taipei 11529, Taiwan.

[4]Graduate Institute of Biotechnology, National Chung Hsing University, Taichung 40227, Taiwan.

[5]Department of Medical Research, China Medical University Hospital, China Medical University, No. 91 Hsueh-Shih Road, Taichung 402, Taiwan.

[6]Biotechnology center, National Chung Hsing University, Taichung 40227, Taiwan.

[7]Department of Ecology and Evolution, University of Chicago, Chicago 60637, USA.

*Corresponding authors address: Biodiversity Research Center, Academia Sinica, 128 Academia Road, Sec. 2, Nankang, Taipei 115, Taiwan.

Corresponding authors E-mail address:

whli@uchicago.edu (W-H Li) and lancecjj@gmail.com (J-J Chang)

Table S1. The minimal inhibitory concentration (MIC) tested in *R. glutinis*.

Medium	Antibiotic	μg/ml	1	10^{-1}	10^{-2}	10^{-3}	10^{-4}	10^{-5}	10^{-6}	10^{-7}
YP2D	-	-	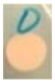	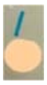	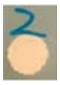	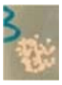	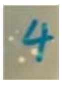	-	-	-
YP2D	Zeocin	100	-	-	-	-	-	-	-	-
YP2D	Zeocin	200	-	-	-	-	-	-	-	-
YP2D	G418	100	-	-	-	-	-	-	-	-
YP2D	G418	200	-	-	-	-	-	-	-	-
YP2D	Hygromycin	100	-	-	-	-	-	-	-	-
YP2D	Hygromycin	200	-	-	-	-	-	-	-	-

* Number 1 to 10^{-7} represented dilution of *R. glutinis* culture fluid in 10 μl drop.

Table S2. The primers used in *R. glutinis* genotype validation.

No.	Primer name	Sequence (5'-3')
1	crtE-13F	AACATCCTCACAGCAATTCCACTCGAGTTT
2	crtE-1065R	TCCAATTTGACGTTCTTCCCCGTTCTTCCT
3	crtI-92F	AAGGTTTCCAGGTCACGGTGTTCGAGAAGA
4	crtI-1551R	TCTGGAGCTGGGGATCGGTCAAAGGATTCG
5	crtYB-26F	TCCATCTGATCTATACTCTCCCAATTCTTG
6	crtYB-1817R	GGTCAATTCCCTTATAAGAATGTTTGGCAA
7	tHMG1-64F	TCCAACCACAATGAAAAACAATCACCATCC
8	tHMG1-1410R	AGCGATAACTCACCGGCCATCACCGCA
9	CBHI-26F	AGACTCACCCGCCTCTGACATGGCAGAAAT
10	CBHI-1438R	AGGACCTGGCAAGTTGTGCCGCTGGCGCA
11	CBHII-90F	ATACTACGCTCAATGTACCCCAGCTGCCGG
12	CBHII-1062R	TTACACCAGTCACCCCAAGCTTGTTGACCA
13	EgIII-110F	GGCCACCATCCGGTCACGAATTGTGCTCCT
14	EgIII-1106R	CAGTCGGTGTTTCCGTCAGGACATACGTGC
15	EgI-47F	CCGCTCTCCCCAGCCGCCAGATGAAGAAGC
16	EgI-1495R	GTCGTTCTGGGCCTTGCAGGTGTAGGGGCT
17	EglA-103F	ACCGGCAGCCAGTGTGTATATGTCGACAAA
18	EglA-576R	TGGCGGGAAAGCCTTGGTTCTGAGTGAGAT
19	BGS-300F	TATTAACTTCGCTCTTGCTCCATCTGTAGG
20	BGS-1960R	TTCAGCGTCAACATCATAGTAAGAAAGATC
21	KanMx-69-F	GTATAAATGGGCTCGCGATAATGTCGGGCA
22	KanMx-712-R	AAAACTCACCGAGGCAGTTCCATAGGATGG
23	ICL-F	TTCCCTTTTTTATACCTTTT
24	crtI-F	TGGGAAAAGAACAAGATCAG
25	crtI-R	CAGAAAGCAAGAACACCAACGGAT

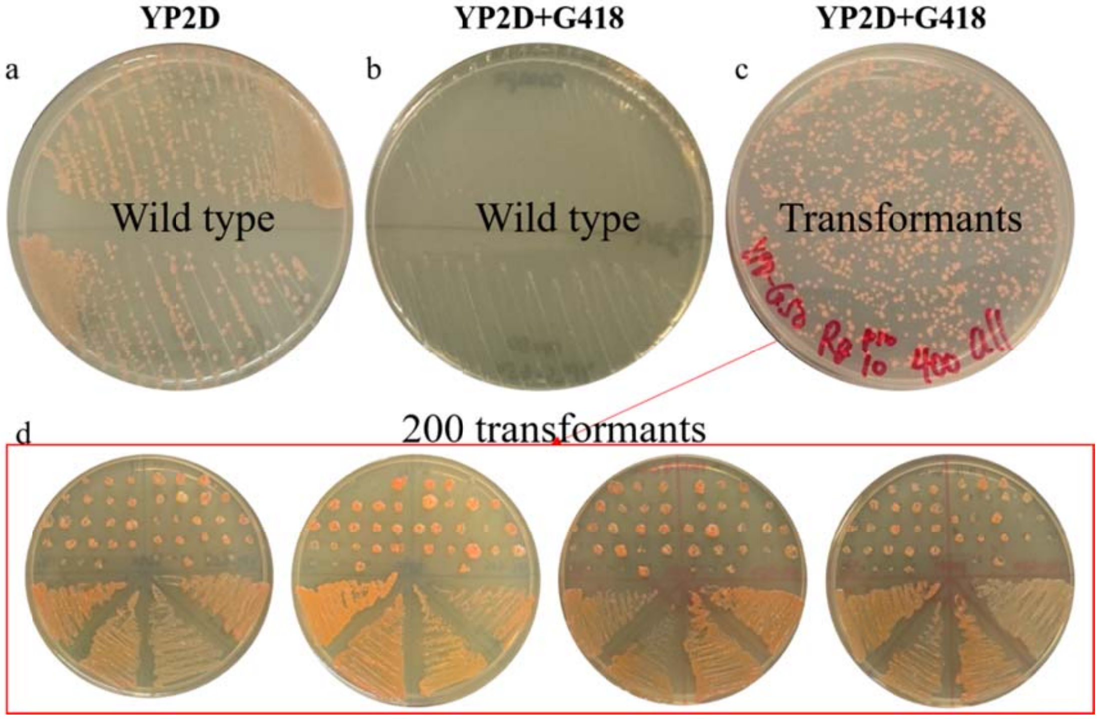

Figure S1. *R. glutinis* transformants on the G418 selective YP2D. *R. glutinis* wild type grew on YP2D, without G418 (a) and with G418 (b). (c) Transformants with *KanMx* gene can grow on the G418 selective YP2D and (d) stably subculture for 3 generations.

(a) *crtI* gene, primers 23, 24 and 25.

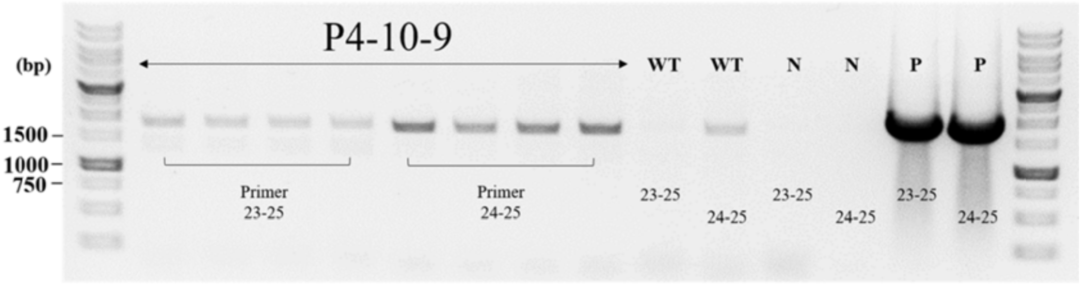

(b) *cbhI* gene, primers 9 and 10.

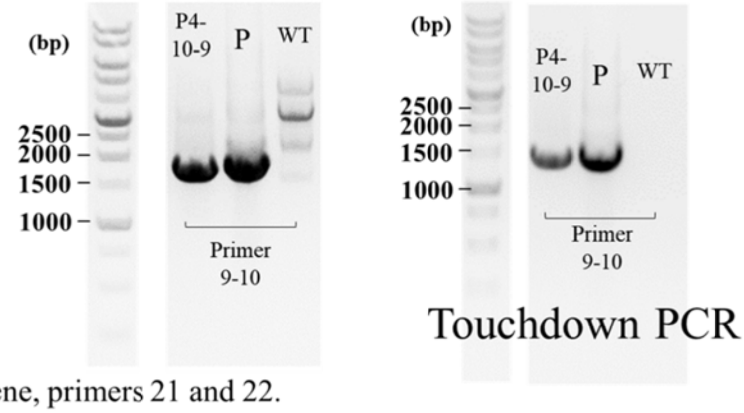

(c) *KanMx* gene, primers 21 and 22.

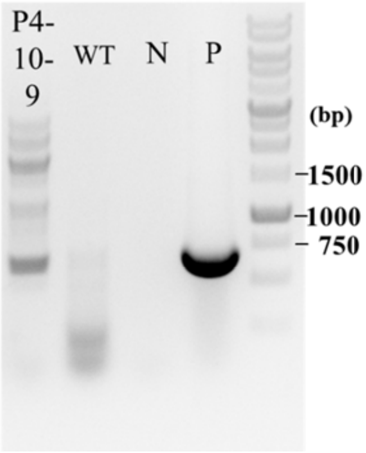

Figure S2. Genotype validating of transformants P4-10-9 by PCR amplification. (a) The primers 23 and 25 can amplify from the ICL promoter region to the end of *crtI* gene; the primers 24 and 25 can amplify the *crtI* gene. (b) The designer 30 bp long primers and touchdown PCR can amplify the correct *cbhI* gene. (c) The designer 30 bp long primers can amplify the correct *KanMx* gene.

(a)

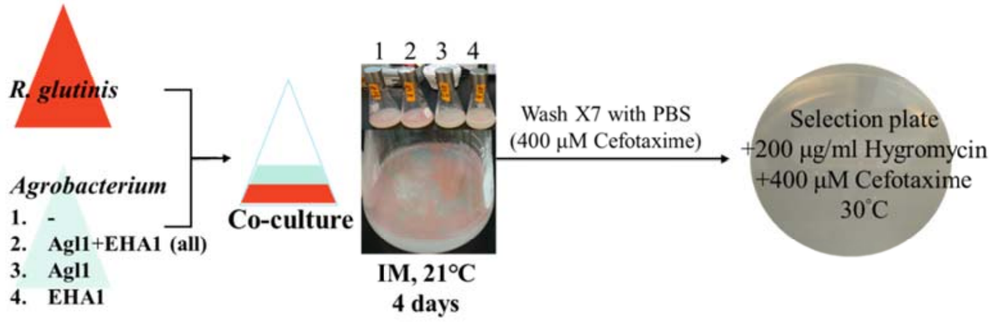

(b)

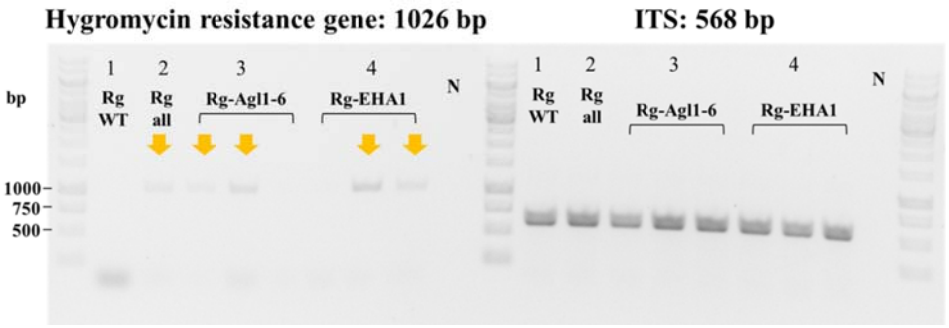

Figure S3.

(a) The *Agrobacterium*-mediated transformation (ATMT) system was applied to transform *R. glutinis* (Lin et al., 2014). Different *Agrobacterium* stains (Agl1 and EHA1) were tested. (b) Genotype validation of transformants by PCR amplification. Hygromycin resistance gene was integrated into the *R. glutinis* chromosome. Internal transcribed spacer (ITS) was used to confirm the *R. glutinis* species.

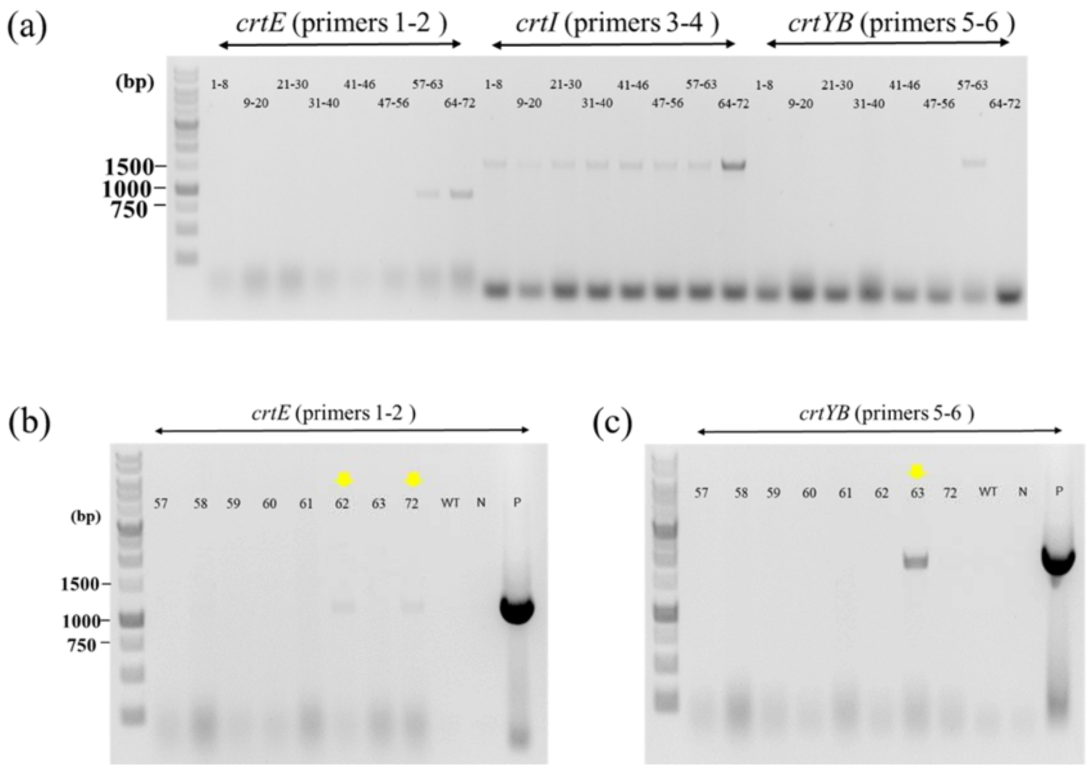

Figure S4. Genotype validation of 2nd time transformants by PCR amplification. (a) The mix colonies PCR showed that 57-72 transformants contained the *crtE* gene and 57-63 transformants contained the *crtYB* gene. (b) The single colony PCR showed that transformants 62 and 72 contained the *crtE* gene. (c) The single colony PCR showed that transformants 63 contained the *crtYB* gene.

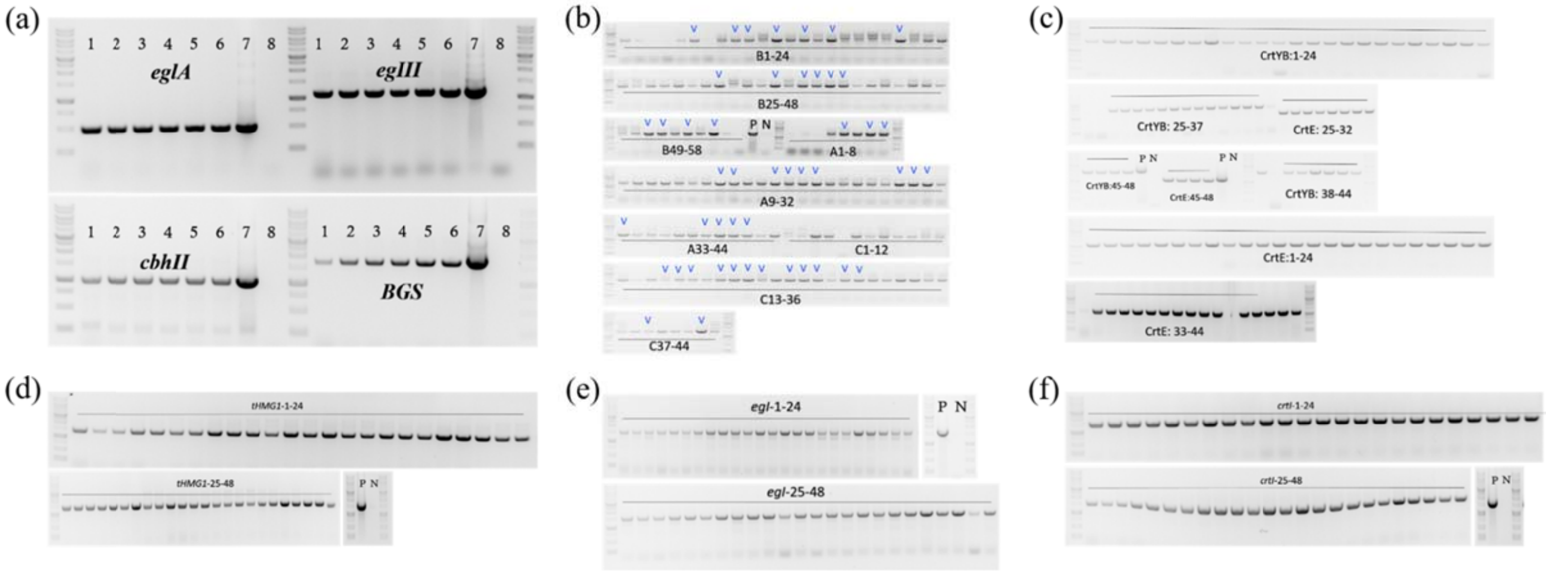

Figure S5. Genotype validation of candida transformants by PCR amplification.

(a) To confirm *EgIII*, *EglA*, *CBHII* and *BGS* genes. The number represented mix colonies (1:1A to 25A, 2: 26A to 44A and 1B to 5B, 3: 6B to 31B, 4: 32B to 58B, 5: 1C to 25C, 6: 26C to 44C, 7: Positive (cassette), 8: Negative (QE)).

(b) To confirm *CBHI* gene. The marker represented the single colony that contained the correct *CBHI* gene size for further genotype validation.

(c) To confirm *crtE* and *crtYB* genes.

(d) To confirm *tHMG1* gene.

(e) To confirm *EgI* gene.

(f) To confirm *crtI* gene.

The 48 colonies were selected because they all contained *EgIII*, *EglA*, *CBHII, BGS*, *CBHI*, *crtE*, *crtYB, tHMG1*, *EgI* and *crtI* genes.

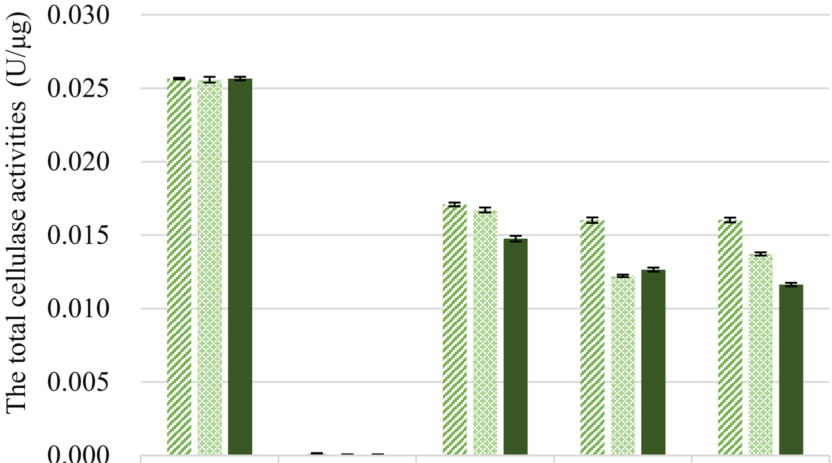

	Tr	WT-3	14B	23C	27C
Sigmacell cellulose type 20	0.026	0.000	0.017	0.016	0.016
Sigmacell cellulose type 50	0.026	0.000	0.017	0.012	0.014
Cellulose microcrystalline	0.026	0.000	0.015	0.013	0.012

Figure S6. Total cellulase activity assay of *R. glutinis* wild type, P4-10-9-63Y-14B, -23C and -27C. The 0.82U commercial enzyme Celluclast® 1.5L applied as standard. All samples used 32 μg of total protein to conduct the assay.

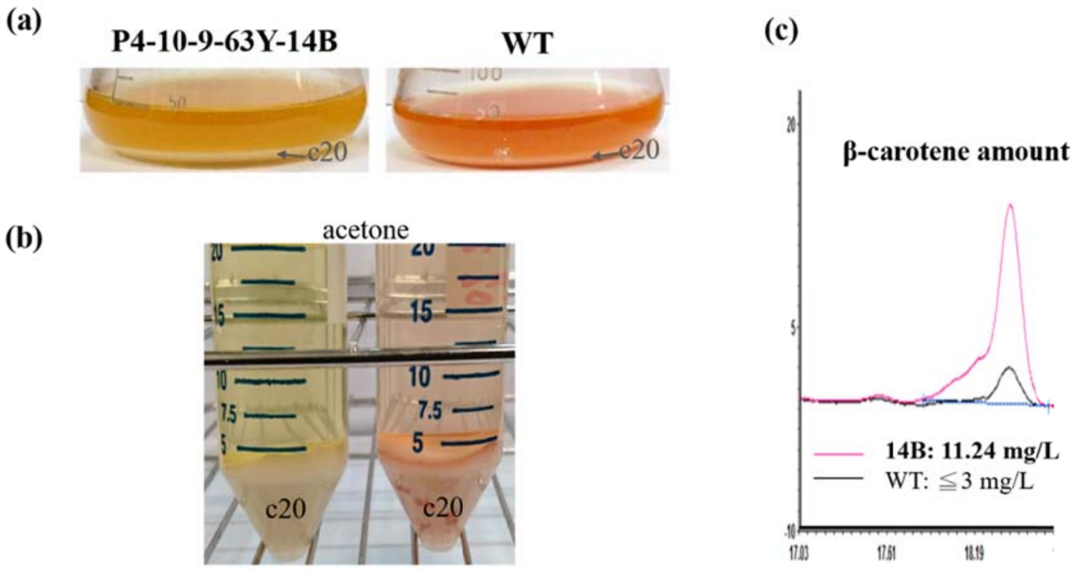

Figure S7. Compared the amounts of β-carotene between *R. glutinis* transformants P4-10-9-63Y-14B and wild type in 50 ml of YP medium with 0.5% glycerol and 2% sigmacell cellulose type 20 (c20).

(a) Colonies in the medium and the c20 substrate. (b) The 50 ml acetone extraction. (c) HPLC results.

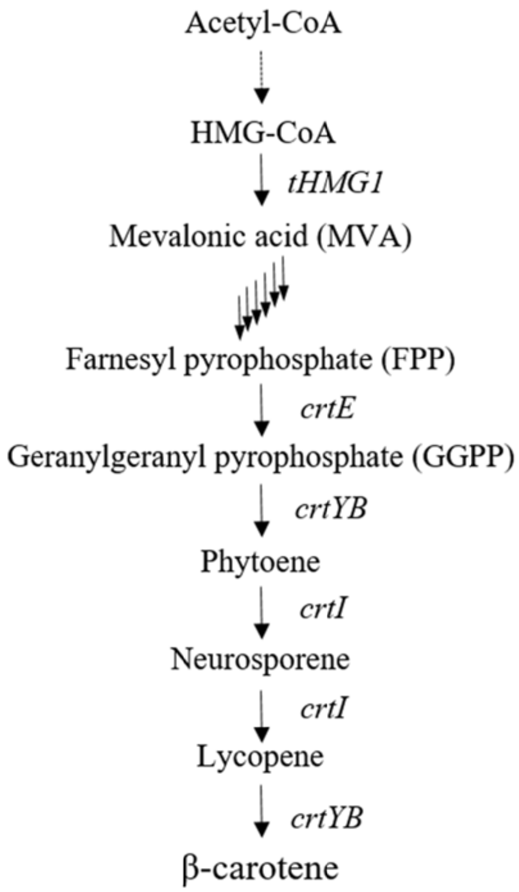

Figure S8. Engineered β-carotene biosynthetic pathway in *R. glutinis*. This pathway shows β-carotene biosynthesis from the Acetyl-CoA in *R. glutinis* (Kot *et al*., 2016).
