# Supplementary material for: Engineering the oleaginous red yeast Rhodotorula glutinis for simultaneous β-carotene and cellulase production
Source: Sci Rep. 2018 Jul 18;8:10850. doi: 10.1038/s41598-018-29194-z (PMC6052021; doi:10.1038/s41598-018-29194-z)
Supplement: Supplementary file 2 — Supplementary raw data [file 41598_2018_29194_MOESM2_ESM.pdf]

Supplementary raw data (Figure)

Engineering the oleaginous red yeast *Rhodotorula glutinis* for simultaneous β-carotene and cellulase production

Hong-Wei Pi[1, 2], Marimuthu Anandharaj[2, 3, 4], Yi-Ying Kao[2], Yu-Ju Lin[2], Jui-Jen Chang[5, *], Wen-Hsiung Li[2, 6, 7, *]

Affiliations:

[1]Ph.D. Program in Microbial Genomics, National Chung Hsing University and Academia Sinica, Taiwan.

[2]Biodiversity Research Center, Academia Sinica, Nankang, Taipei 11529, Taiwan.

[3]Molecular and Biological Agricultural Sciences Program, Taiwan International Graduate Program, National Chung Hsing University and Academia Sinica, Taipei 11529, Taiwan.

[4]Graduate Institute of Biotechnology, National Chung Hsing University, Taichung 40227, Taiwan.

[5]Department of Medical Research, China Medical University Hospital, China Medical University, No. 91 Hsueh-Shih Road, Taichung 402, Taiwan.

[6]Biotechnology center, National Chung Hsing University, Taichung 40227, Taiwan.

[7]Department of Ecology and Evolution, University of Chicago, Chicago 60637, USA.

*Corresponding authors address: Biodiversity Research Center, Academia Sinica, 128 Academia Road, Sec. 2, Nankang, Taipei 115, Taiwan.

Corresponding authors E-mail address:

whli@uchicago.edu; whli@gate.sinica.edu.tw (W-H Li) and lancecjj@gmail.com (J-J Chang)

Supplementary Information:

Supplementary raw data (Figure)

The raw figures used in making Figures 5, Supplementary Table S1 and other related Supplementary Figures. The way we generated the figures from raw images were labeled in red.

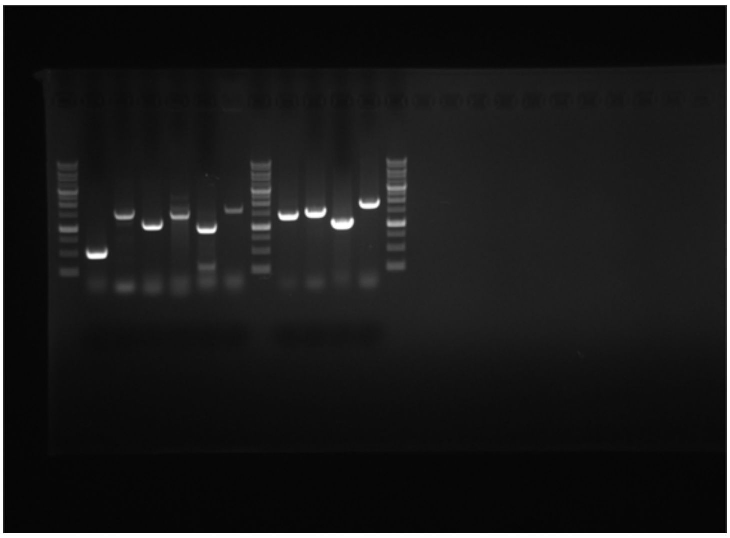

P4-10-9-63Y-14B

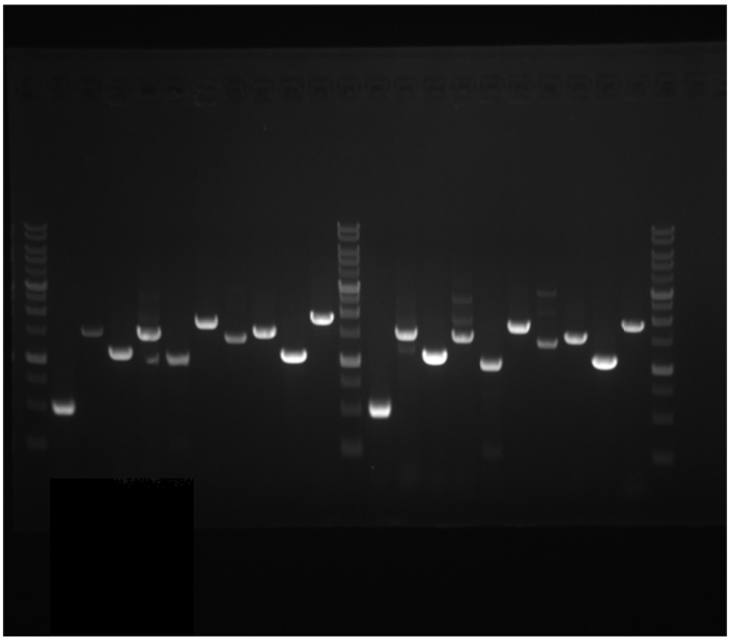

P4-10-9-63Y-23C and -27C

The raw images used in making Fig. 5. To make the Fig. 5 in the main text, we cut the gel image and combine these two gel images together.

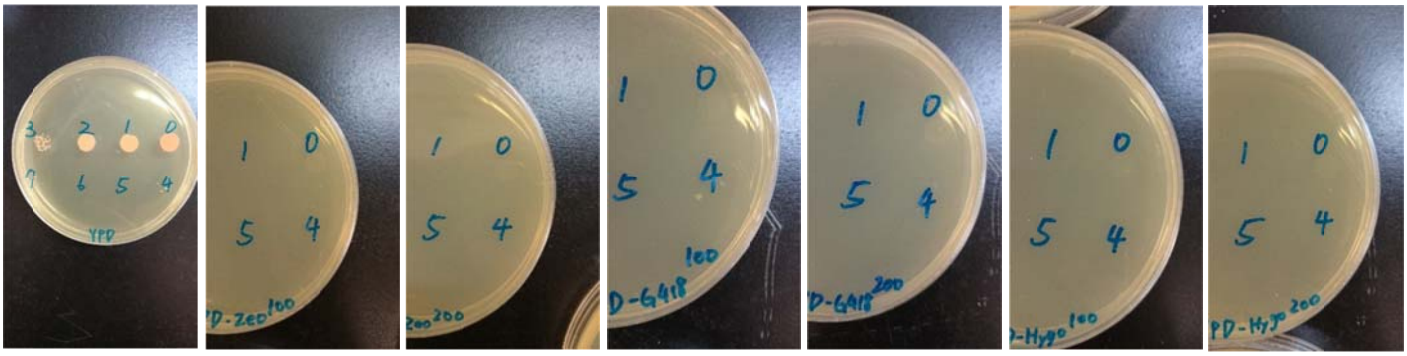

The raw images used in making Supplementary Table S1. To make the Supplementary Table S1 in the Supplementary Information file, we cut the colonies image and reorganizes these images together as table. There were no colony growing in either 100 or 200 µg/ml concentration of any of the three antibiotics plates so we did not put the images.

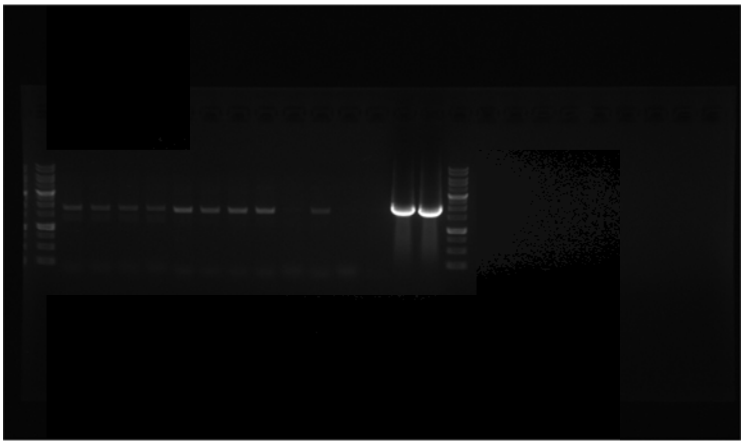

Supplementary Figure S2a raw image

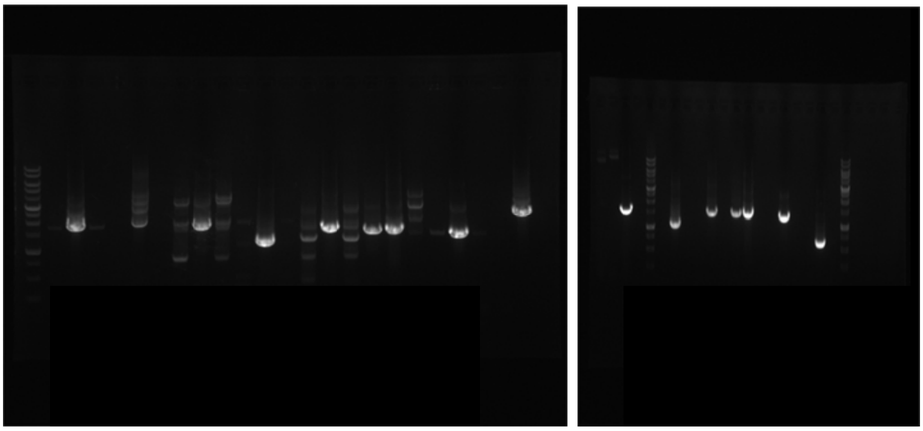

Supplementary Figure S2b raw image

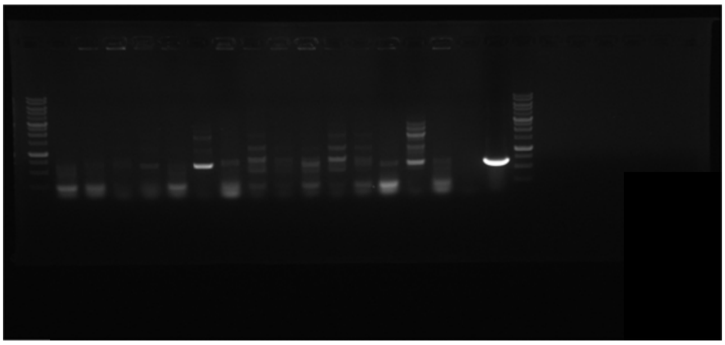

Supplementary Figure S2c raw image

The raw images used in making Supplementary Fig. S2. To make the Supplementary Fig. S2 in the Supplementary Information file, we cut the gel image, inverted color and combine these gel images together.

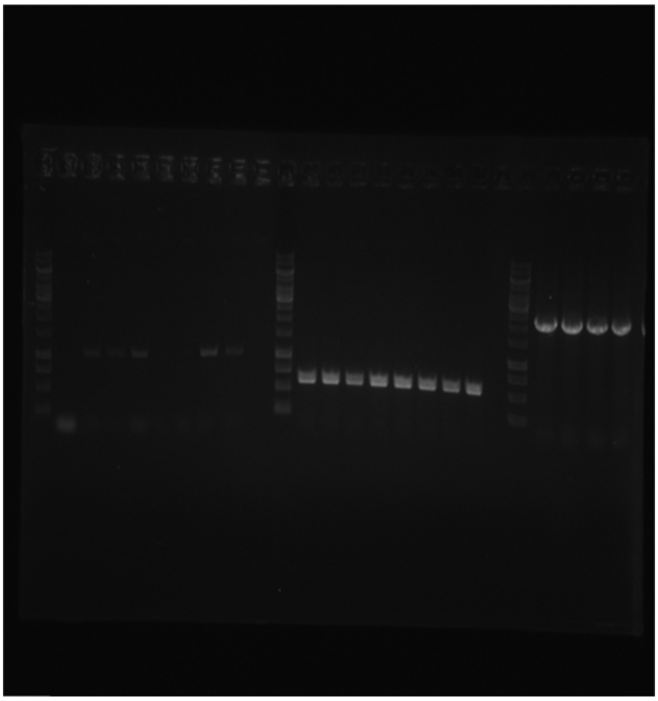

The raw images used in making Supplementary Fig. S3. To make the Supplementary Fig. S3b in the Supplementary Information file, we cut the gel image and inverted color.

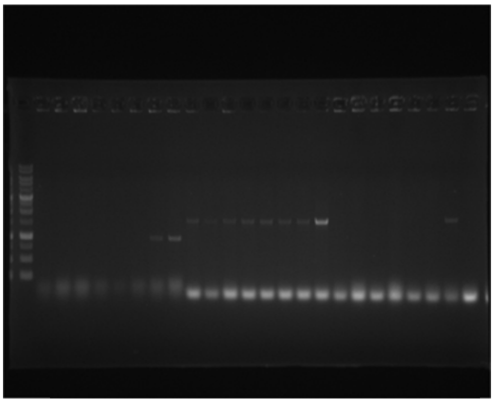

Supplementary Figure S4a raw image

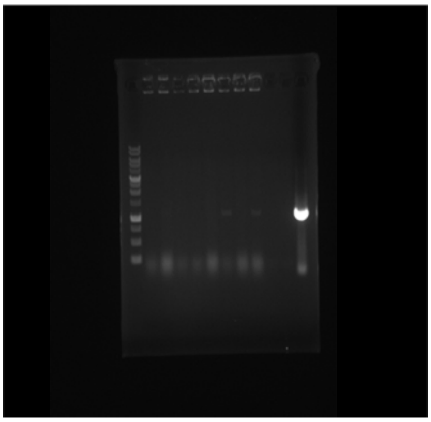

Supplementary Figure S4b raw image

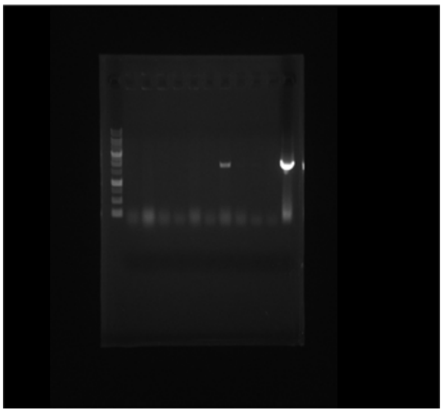

Supplementary Figure S4c raw image

The raw images used in making Supplementary Fig. S4. To make the Supplementary Fig. S4 in the Supplementary Information file, we cut the gel image, inverted color and combine these gel images together.

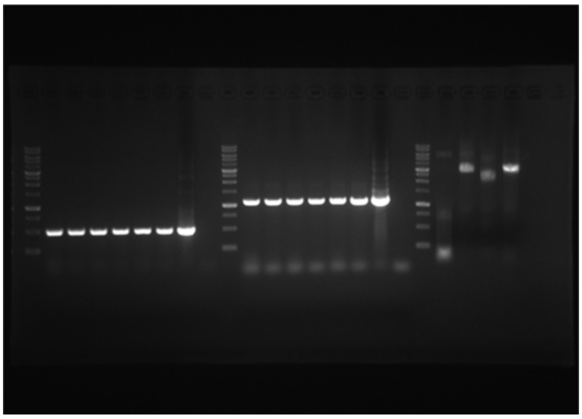

eglA and *egIII*

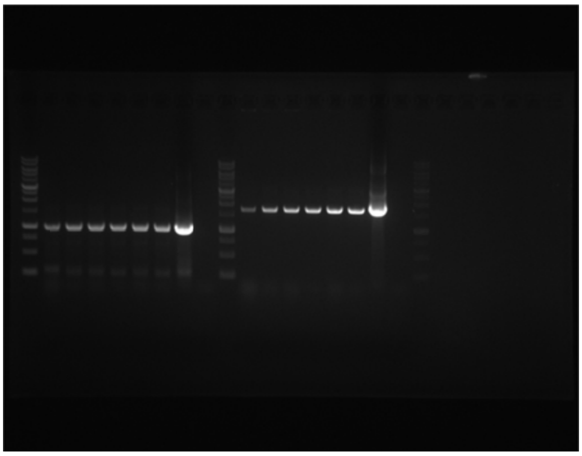

CBHII and *BGS*

The raw images used in making Supplementary Fig. S5a. To make the Supplementary Fig. S5a in the Supplementary Information file, we cut the gel image, inverted color and combine these gel images together.

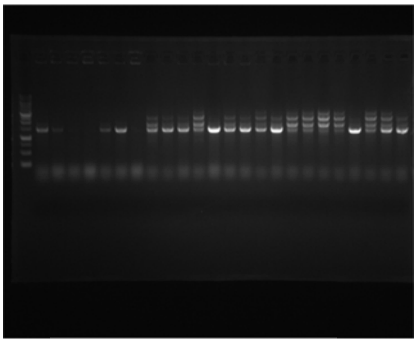

cbhI (B1-24)

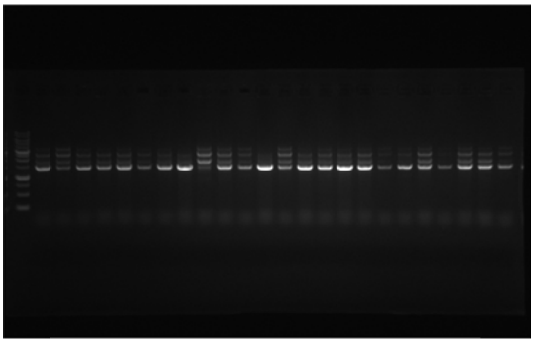

cbhI (B25-48)

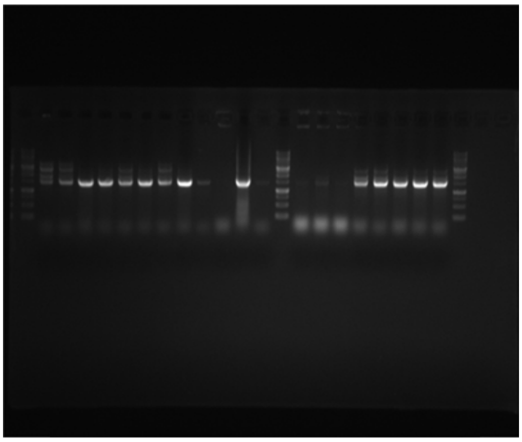

cbhI (B49-58 and A1-8)

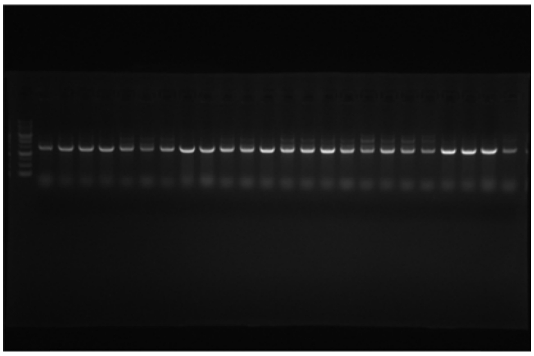

cbhI (A9-32)

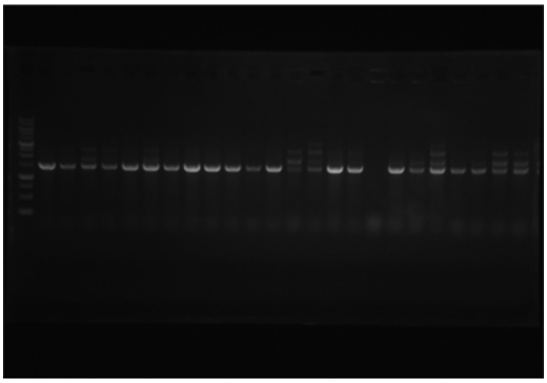

cbhI (A33-44 and C1-12)

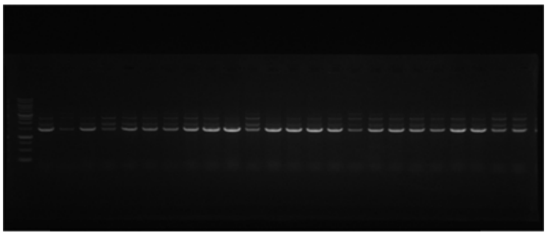

cbhI (C13-36)

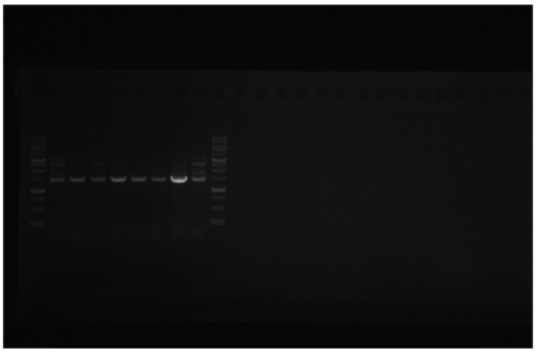

cbhI (C37-44)

The raw images used in making Supplementary Fig. S5b. To make the Supplementary Fig. S5b in the Supplementary Information file, we cut the gel image, inverted color and combine these gel images together.

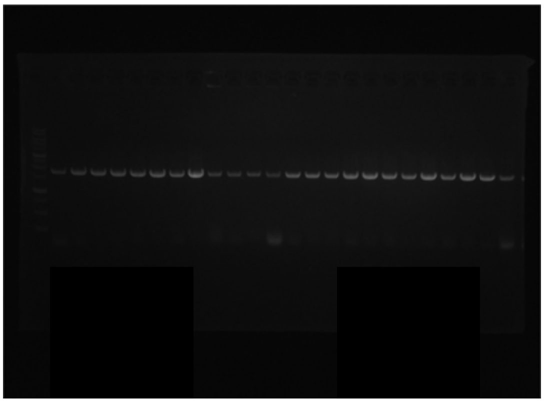

crtYB (1-24)

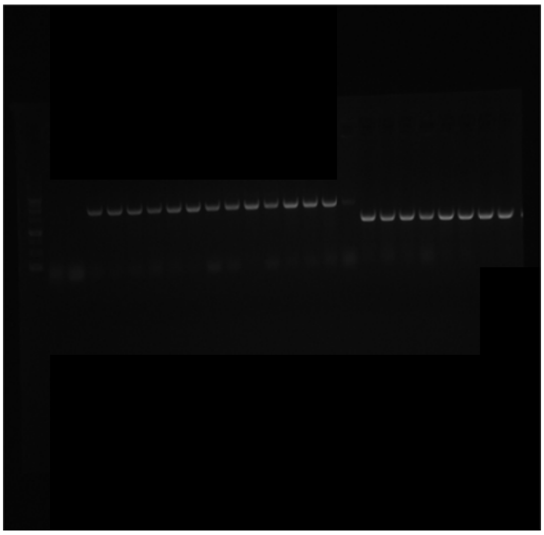

crtYB (25-37) and *crtE* (25-32)

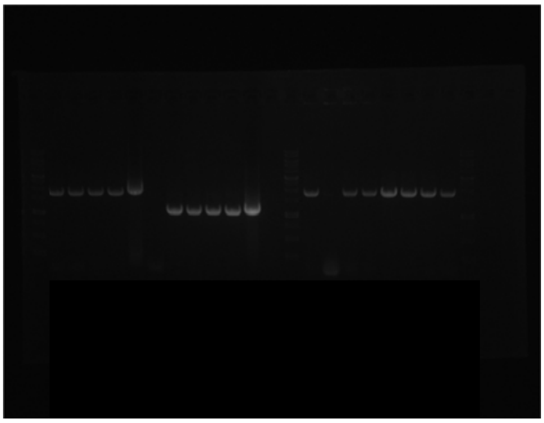

crtYB (38-48) and *crtE* (45-48)

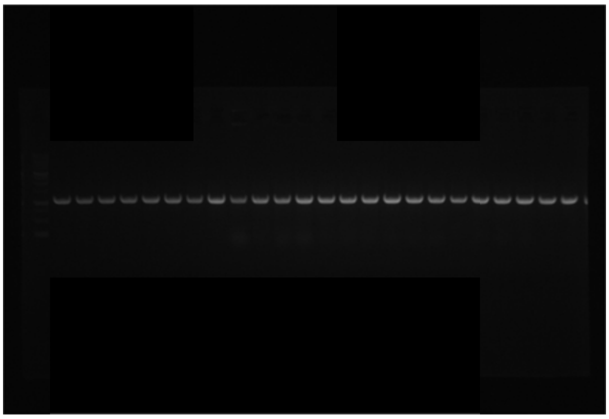

crtE (1-24)

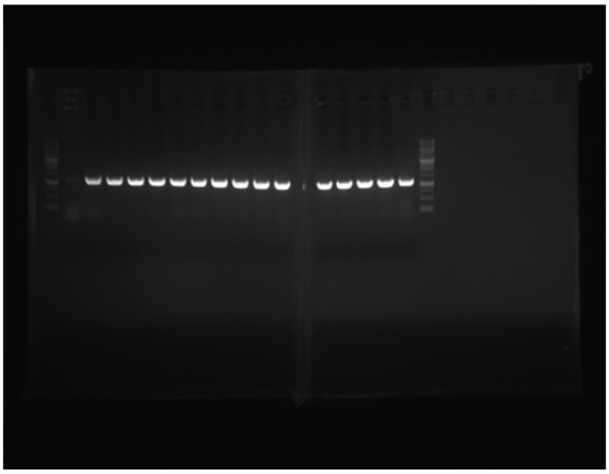

crtE (33-44)

The raw images used in making Supplementary Fig. S5c. To make the Supplementary Fig. S5c in the Supplementary Information file, we cut the gel image, inverted color and combine these gel images together.

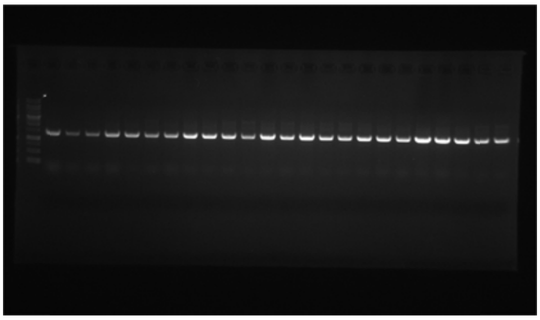

tHMG1 (1-24)

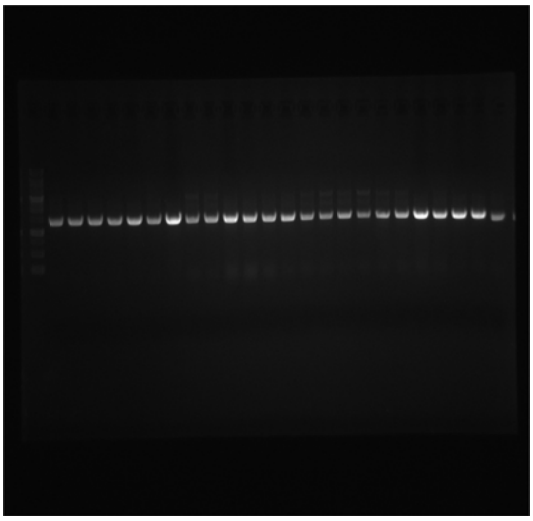

tHMG1 (25-48)

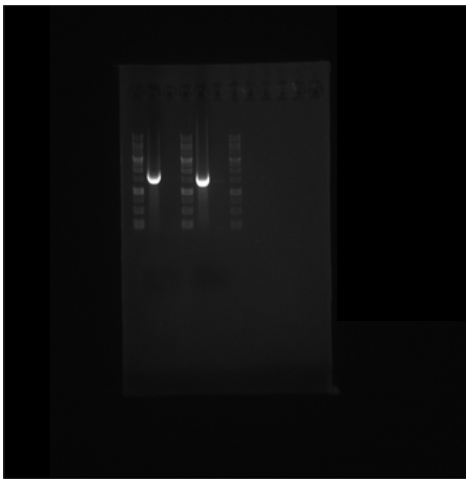

crtI and *tHMG1* (positive and negative control)

The raw images used in making Supplementary Fig. S5d. To make the Supplementary Fig. S5d in the Supplementary Information file, we cut the gel image, inverted color and combine these gel images together.

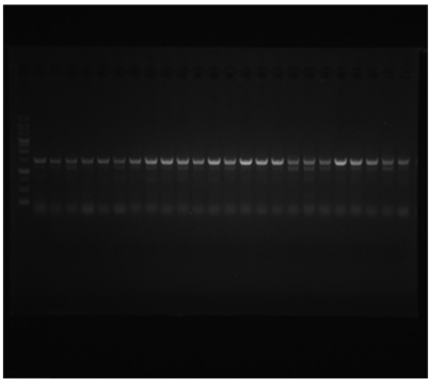

egI (1-24)

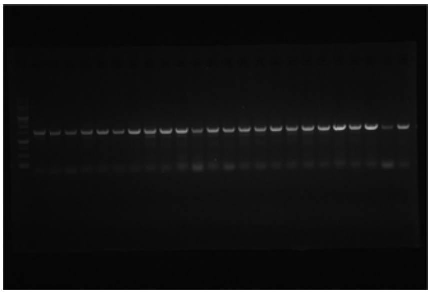

egI (25-48)

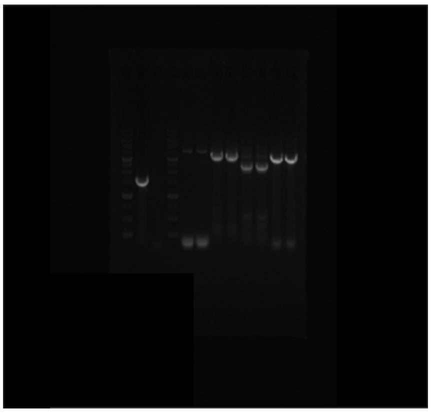

egI (positive and negative control)

The raw images used in making Supplementary Fig. S5e. To make the Supplementary Fig. S5e in the Supplementary Information file, we cut the gel image, inverted color and combine these gel images together.

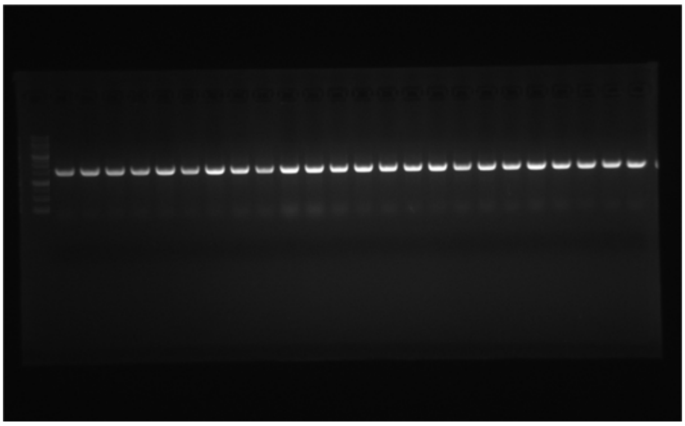

crtI (1-24)

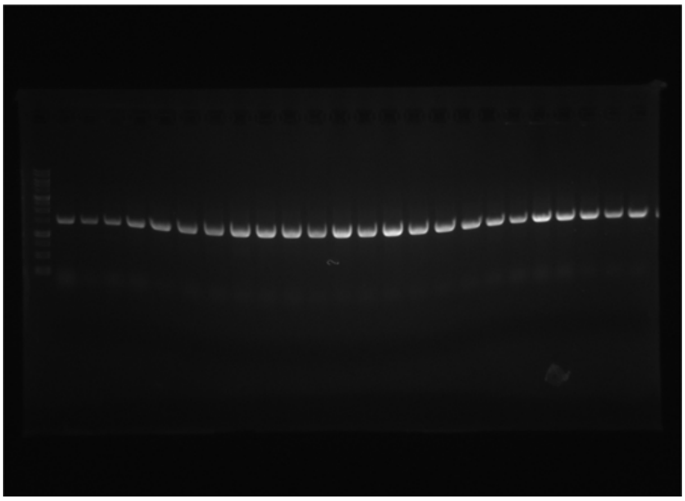

crtI (25-48)

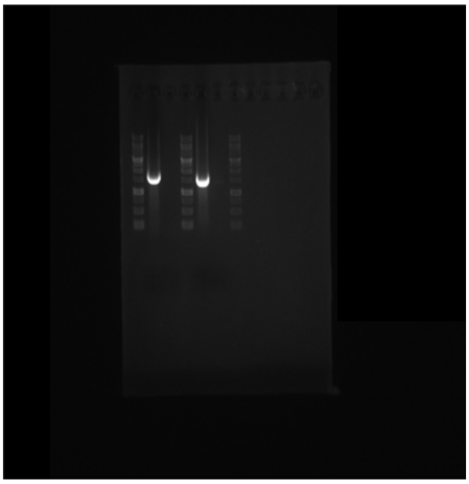

crtI and *tHMG1* (positive and negative control)

The raw images used in making Supplementary Fig. S5f. To make the Supplementary Fig. S5f in the Supplementary Information file, we cut the gel image, inverted color and combine these gel images together.
